# Inhibiting the Cholesterol Storage Enzyme ACAT1/SOAT1 in Myelin Debris-Treated Microglial Cell Lines Activates the Gene Expression of Cholesterol Efflux Transporter ABCA1

**DOI:** 10.3390/biom14101301

**Published:** 2024-10-14

**Authors:** Thao N. Huynh, Matthew C. Havrda, George J. Zanazzi, Catherine C. Y. Chang, Ta Yuan Chang

**Affiliations:** 1 Department of Biochemistry and Cell Biology, Geisel School of Medicine at Dartmouth, Hanover, NH 03755, USA; thao.n.huynh.gr@dartmouth.edu; 2 Department of Molecular and System Biology, Geisel School of Medicine at Dartmouth, Hanover, NH 03755, USA; matthew.c.havrda@dartmouth.edu; 3 Department of Pathology and Laboratory Medicine, Dartmouth–Hitchcock Medical Center, Lebanon, NH 03766, USA; george.j.zanazzi@dartmouth.edu

**Keywords:** Alzheimer’s disease, myelin debris, microglia, cholesterol, cholesteryl esters, acyl-CoA:cholesterol acyltransferase, sterol O-acyltransferase, ACAT inhibitor, ATP-binding cassette subfamily A member 1, aging, foam cell, liver X receptor

## Abstract

Aging is the major risk factor for Alzheimer’s disease (AD). In the aged brain, myelin debris accumulates and is cleared by microglia. Phagocytosed myelin debris increases neutral lipid droplet content in microglia. Neutral lipids include cholesteryl esters (CE) and triacylglycerol (TAG). To examine the effects of myelin debris on neutral lipid content in microglia, we added myelin debris to human HMC3 and mouse N9 cells. The results obtained when using ^3^H-oleate as a precursor in intact cells reveal that myelin debris significantly increases the biosynthesis of CE but not TAG. Mass analyses have shown that myelin debris increases both CE and TAG. The increase in CE biosynthesis was abolished using inhibitors of the cholesterol storage enzyme acyl-CoA:cholesterol acyltransferase 1 (ACAT1/SOAT1). ACAT1 inhibitors are promising drug candidates for AD treatment. In myelin debris-loaded microglia, treatment with two different ACAT1 inhibitors, K604 and F12511, increased the mRNA and protein content of ATP-binding cassette subfamily A1 (ABCA1), a protein that is located at the plasma membrane and which controls cellular cholesterol disposal. The effect of the ACAT1 inhibitor on ABCA1 was abolished by preincubating cells with the liver X receptor (LXR) antagonist GSK2033. We conclude that ACAT1 inhibitors prevent the accumulation of cholesterol and CE in myelin debris-treated microglia by activating ABCA1 gene expression via the LXR pathway.

## 1. Introduction

Myelin is a cholesterol-rich material that forms the insulating sheath around nerve cells [1]. Myelin contains up to 70–80% of the total brain cholesterol in the adult brain [2]. Proper myelination is crucial for synaptic transmission and metabolic support in neuronal cells [1]. Myelin debris results from the breakdown of myelin [3] and accumulates in the aging brain and in certain neurodegenerative diseases [4,5]. Under normal conditions, microglia and other phagocytes clear myelin debris to enable proper remyelination [4]. However, in aging mouse models, myelin debris accumulates inside the microglia [4]. When microglia become overloaded with myelin debris, the cells experience a pro-inflammatory response, which may further contribute to cognitive decline in aging and neurodegenerative diseases [4,6]. Myelin debris-overloaded microglia often exhibit a “foamy” phenotype, characterized by the accumulation of neutral lipid droplets [7,8,9]. Neutral lipid droplets consist mainly of cholesteryl esters (CE) and triacylglycerol (TAG) [10].

Aging is the major risk factor for Alzheimer’s disease (AD). While animal models for aging are becoming available [11], cell models for aging are sparse. Microglia play important roles in the pathogenesis of AD [9,12,13,14,15]. Myelin debris-treated microglia have been considered as cell models for mammalian cell aging. For example, a previous study by Nugent et al. showed that treatment of myelin debris in human iPSC microglia and in mouse primary microglia caused CE accumulation, while inhibition of the cholesterol storage enzyme acyl-CoA:cholesterol acyltransferase, also known as sterol O-acyltransferase 1 (ACAT1/SOAT1), reduced CE accumulation, demonstrating that myelin debris stimulates CE biosynthesis [7]. However, the downstream effects of inhibiting ACAT1/SOAT1 in myelin debris-treated microglia have not been well examined. Altering ACAT1 activity in these cells is likely to affect cholesterol homeostasis through its interacting with other key players involved in regulating cellular lipid metabolism [16,17]. Liver X receptors (LXRs) are nuclear receptors that regulate cholesterol homeostasis, lipid metabolism and membrane phospholipid composition and they play important roles in immune responses [18,19,20]. The natural ligands for LXR are oxysterols [21]. In a mouse model for tauopathy, Litvinchuk et al. [22] have shown that a synthetic LXR agonist, GW3965, increases gene expression of ABCA1, which is the major cholesterol efflux protein that controls cellular cholesterol disposal [23]. Additional results by Litvinchuk et al. show that overexpression of the ABCA1 gene also reduces CEs and suppresses tauopathy [22]. This study suggests that interventions to stimulate cholesterol efflux from microglia via activating ABCA1 to defend against AD warrant further investigation. Gouna et al. have also shown that treating wild-type mouse microglia with myelin debris causes CE accumulation [24]. In these studies, whether myelin debris also affects TAG biosynthesis is unclear. To address these issues, in the current work, we use two cell lines, mouse N9 and human HMC3 microglia, as our cell models. These cell lines have been used successfully by other investigators as microglia cell models for AD research [25,26,27,28,29,30,31]. We prepared myelin debris from both mouse and human brains, and then added them to these cells to monitor their effects on CE and TAG biosynthesis. We also studied the downstream effects of the ACAT1 inhibitor in these cells.

## 2. Materials and Methods

### 2.1. Animals

Wild-type (WT) mice in C57B6/J background were obtained from Jackson Laboratory (Bar Harbor, ME, USA). Mice that were 3–6 months old were used for myelin debris isolation. All mouse procedures were approved by the Dartmouth Institutional Animal Care and Use Committee.

### 2.2. Human Brain Samples

Human brain samples were donated by the Anatomy Gifts Registry (Hanover, MD, USA) and the Department of Pathology and Laboratory Medicine at Dartmouth–Hitchcock Medical Center (Lebanon, NH, USA). All brain samples were received frozen prior to their use.

### 2.3. Cell Culture

Both the N9 and HMC3 (ATCC^®^CRL-3304) cell lines were obtained from ATCC. All cells were maintained at 37 °C with 5% CO_2_ in a humidified incubator. Mouse N9 microglial cells were maintained in RPMI-1640 with 10% heat-inactivated serum. Human HMC3 microglial cells were maintained in MEM supplemented with 10% heat-inactivated serum and 1% non-essential amino acids. HEK-293 cells were maintained in DMEM with 10% serum.

The ACAT1/SOAT1 inhibitors K604 and F12511 were first dissolved in DMSO at 5 mM as stock solutions and then diluted into the culture medium such that the final concentration was 0.5 µM, as previously described in [16,32,33,34]. For LXR agonist treatment, T0901317 was diluted to a final concentration of 10 µM in complete media as described above and added to the cells for 24 h, as previously described in [32,35]. For LXR antagonist treatment, GSK2033 was diluted to a final concentration of 5 µM in complete media and added to cells for 24 h, as previously described in [36].

### 2.4. Myelin Isolation and Characterization of Myelin Debris

Myelin debris were isolated from mouse and human frozen brain samples following a protocol adapted from [37], with minor modifications. Briefly, brain samples were minced and homogenized in 0.3M sucrose in Tris buffer (20 mM Tris-HCl, pH 7.45, 1mM EDTA with protease inhibitor cocktail), using an automatic Dounce chamber for 20 strokes. The homogenates were layered on top of a 0.83M sucrose solution and spun at 75,000× *g* for 70 min with minimum acceleration and deceleration. Cloudy myelin layers at the interface were collected and subjected to two rounds of osmotic shock by adding Tris buffer, followed by centrifugation at 75,000× *g* for 70 min with minimum acceleration and deceleration. Myelin debris, post osmotic shock and pelleted at the bottom of the tube, were resuspended in MilliQ water and spun at 15,000× *g* for 20 min to obtain clean myelin debris for cell treatment.

Myelin debris protein content was determined by Lowry protein assay, and cholesterol content was measured following the manufacturer’s protocol using the Wako Free Cholesterol Kit E. For both mouse and human myelin debris preparations, the ratio of total protein to cholesterol was 2:1 (µg protein to µg cholesterol). This value is consistent with the value reported by [3]. Protein content characterization of human and mice myelin debris was analyzed by running a 15% SDS-PAGE gel and staining with colloidal Coomassie blue dye.

### 2.5. Intact Cell ^3^H-Oleate Pulse

^3^H-oleate pulse in intact cells was performed according to a procedure previously described in [38,39]. ^3^H-oleate enters the cell interior and forms oleyl coenzyme A through acyl coenzyme A synthetases [40]. Oleyl coenzyme A then serves as a common precursor for CE biosynthesis via ACATs, and for TAG biosynthesis via diacylglycerol acyltransferases (DGATs) [41,42].

Mouse N9 and human HMC3 microglia were treated for 24 h with various concentrations of either control (DMSO), myelin debris, or the ACAT1 inhibitors K604 or F12511. Following treatment, cells were pulsed for 30 min (N9) or 2 h (HMC3) at 37 °C with ^3^H-oleate/fatty acid-free BSA. Cells were then washed 3 times with ice-cold PBS and lysed with 0.2M NaOH, under orbital shaking for 30 min. Aliquots were withdrawn for protein concentration determination using the Lowry assay. The solubilized cell slurries were neutralized with 3M HCl and 1M KH_2_PO_4_. Nonradioactive CE and TAG, of 40 µg each, were added per sample to aid in identification after TLC analysis. Lipid extractions were performed with CHCl_3_:MeOH (2:1) and water. Samples were vortexed and centrifuged at 500 rpm for 10 min. The top-phase was removed, and the bottom phase was blow dried by N_2_ using an N-evap apparatus. Dried samples were vortexed vigorously with 50 µL of ethyl acetate and spotted on TLC plates. The solvent system used was petroleum ether:ethyl ether:acetic acid (90:10:1). The CE bands (Rf = 0.9) and the TAG bands (Rf = 0.5) were identified after TLC by iodine staining and scraped from the TLC. Radioactivity readouts were measured by scintillation counter.

### 2.6. Nile Red Staining and Image Analysis

Nile Red was used to monitor lipid droplets in live cells according to procedures previously described in [43,44]. Briefly, primary microglia were plated on MaTek (Ashland, MA, USA) 35 mm dishes pre-coated with poly-L-lysine at 2 × 10^6^ cells per plate overnight. Treatment was performed in serum-free DMEM, and cells were rinsed three times with HBSS (Gibco by ThermoFisher, Waltham, MA, USA). Cells were treated with 100 ng/mL Nile Red and incubated for 10 min at 37 °C, 5% CO_2_, protected from light. Cells were rinsed in HBSS and imaged in serum-free MEM, with no phenol red (Gibco by ThermoFisher, Waltham, MA, USA), on the confocal fluorescence microscope. For image analysis, data were collected and analyzed using Fiji-ImageJ software version 2.1.0/1.53c. Briefly, all images were set to the same threshold, fluorescence intensities were measured and normalized against total cell area to obtain a mean fluorescence intensity.

### 2.7. TLC Analysis of Intracellular Cholesterol, CE and TAG Contents

For intracellular cholesterol, CE and TAG content analysis, cells were analyzed following the procedure described in [34,45]. Briefly, cells were treated as indicated in the treatment scheme and lysed by scraping in distilled water. Lipids were then extracted using CHCl_3_:MeOH (2:1) extraction and water, then dried in a similar manner as described in Section 2.5. Dried samples were spotted on a TLC plate and separated using hexanes, ethyl ether: acetic acid (65:35:2). Bands were visualized by iodine staining and quantified on a standard curve produced in the same TLC plate.

### 2.8. Whole Cell Protein Isolation and Western Blot Analyses

For whole cell protein isolation, cells were harvested in 10% SDS for ACAT1 Western blots, or in RIPA buffer for the other Western blots, containing protease inhibitor cocktail (Sigma) and incubated at 4 °C for 30 min. Cell lysates were then centrifuged, and the protein concentration of the supernatant was determined using the Lowry protein assay. For mouse and human myelin debris Western blot, Laemmli sample buffer (supplemented with 350mM dithiothreitol final concentration) was directly added to the isolated myelin debris fraction. The lysates were run on a 6% and 10% SDS-PAGE gel and transferred to a 0.45 μm nitrocellulose membrane for 4 h at 300 mA. After blocking in 5% milk in TBST buffer overnight at 4 °C, the membranes were incubated overnight with anti-ABCA1 (Novus NB400-105), anti-ACAT1 (DM102) produced in the Chang lab, anti-PLIN2 (Proteintech 15294-1-AP), anti-MBP (Novus NB600-717) and anti-vinculin (Millipore 05-386) as a protein loading control. Blots were then washed and incubated with secondary antibodies from Li-Cor appropriate for the species. Western blot images were captured on the Li-Cor Odyssey CLx and analyzed on Li-Cor Image Studio. Analyzed protein bands were within the linear range of detection.

### 2.9. RNA Extraction

For whole cell RNA extraction, cells were lysed in Trizol. Chloroform was added (0.2 mL per ml of Trizol) and the samples were vortexed and spun at 10,000× *g* for 18 min. The top aqueous phase was removed and transferred to a new tube. An equal volume of 100% EtOH was added and 700 µL aliquots were loaded onto a Qiagen RNeasy column. The Qiagen RNeasy Plus Mini Kit was used for all subsequent steps according to the manufacturer’s protocol.

### 2.10. NanoString nCounter Elements XT Assay and Analysis

These experiments were performed using facilities available at the Genomics and Molecular Biology Shared Resources at the Dartmouth Cancer Center. NanoString-recommended protocols were followed, as described in the nCounter Elements XT Assay user’s manual (June 2018). Data collection was performed using an nCounter digital analyzer. An nCounter reporter library file (RLF), specific to the reporter code sets used, was uploaded prior to scanning on the digital analyzer. The nCounter cartridge was loaded into a cartridge carrier at the top of the digital analyzer, which can hold up to six cartridges at a time. The digital analyzer uses epifluorescence microscopy and a CCD camera to yield target molecule counts by imaging each of the 12 channels of the cartridges independently. Digital images were processed within the nCounter instrument, and the reporter probe counts were tabulated in comma-separated value (CSV) format for data analysis. After sample imaging, data were downloaded and imported into NanoString nSolver analysis software version 4.0.

### 2.11. Synthesis of ACAT1/SOAT1 Inhibitors

K604 and F12511 were custom synthesized by WuXi AppTec in China. Based on HPLC-MS and NMR profiles, the purity of K604 was 98%. F12511 was 98% pure in terms of stereospecificity. K604 is a high-affinity, selective ACAT1 with K_i_ = 0.45 μmol/L for ACAT1 and K_i_ = 102.9 μmol/L for ACAT2 [46]. F12511 is another potent ACAT inhibitor that has an affinity for both ACAT1 (K_i_ = 0.039 μmol/L) and ACAT2 (K_i_ = 0.110 μmol/L) [46]. K604 is competitive against oleyl-CoA (K_i_ = 0.378 μmol/L), which is the preferred fatty acyl-CoA substrate for ACAT1 [47]. F12511 is a fatty acid anilide derivative [48,49].

### 2.12. Statistical Analysis

All statistical analyses were performed using Prism10 software version 10.1.0 (GraphPad). A one-way ANOVA test with Sidak correction for multiple comparisons between treatment groups were used. Error bars indicate SEM. * *p* < 0.05; ** *p* < 0.01; *** *p* < 0.001; **** *p* < 0.0001.

## 3. Results

### 3.1. Characterization of Myelin Debris Isolated from Human and Mouse Brain Tissues

**Figure 1:**
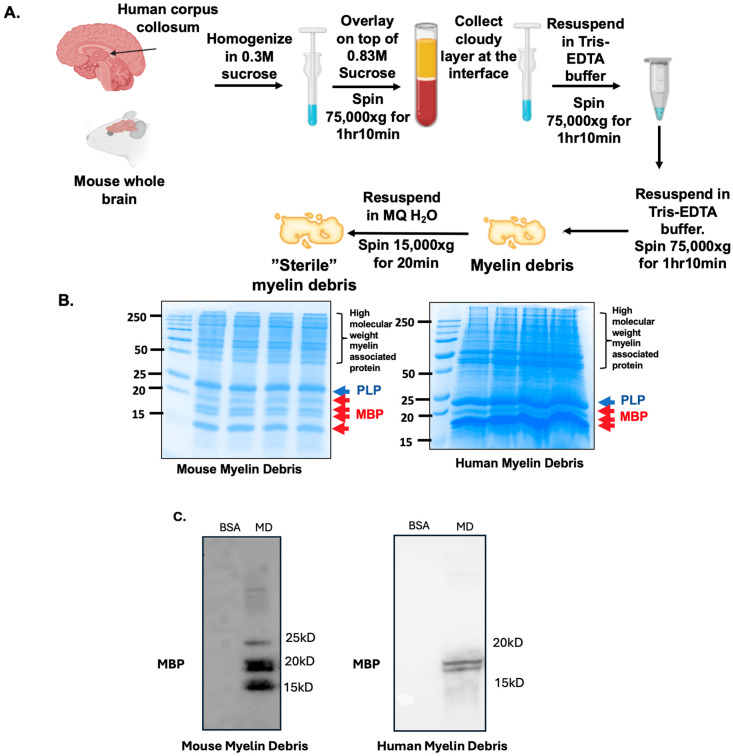
Characterization of mouse and human myelin debris. (**A**) Procedure for crude myelin debris isolation from frozen brain tissue as described in Section 2. Images were created using BioRender. (**B**) Representative colloidal Coomassie blue staining on a 15% SDS-PAGE gel for characterization of the crude myelin debris fraction. Signature protein associated with myelin fraction, such as proteolipid protein (PLP) or myelin basic protein (MBP) are labeled in blue and red, respectively. N = 4. (**C**) Representative Western blot for MBP in isolated human and mouse myelin debris. BSA at equal protein concentration was used as negative control. Western blot original images are in the Supplementary Materials.

To establish a simple system by which to study myelin debris-loaded microglia, we first isolated myelin debris from human and mouse brain samples, following the procedure shown in Figure 1A. The myelin debris fraction was characterized using a 15% SDS-PAGE gel, to identify proteins typically associated with myelin debris, including proteolipid protein (PLP) and myelin basic protein (MBP). PLP provides structural stability for myelin [50], while MBP is responsible for signaling and adhesion on the myelin cytosolic surface [51]. Results in Figure 1B show a migration of dominated protein patterns that is similar to PLP and MBP. These protein patterns are consistent with isolated myelin debris in previously published literature [52]. Additionally, the protein components of myelin debris remain consistent across different batches of isolated myelin (Figure 1B). When comparing human myelin debris from the corpus callosum and mouse myelin debris from whole brain tissue, their protein band patterns on SDS-PAGE are similar. In our isolated myelin debris, PLP and MBP are the two most enriched proteins (Figure 1B). To ensure that our isolated myelin debris is enriched in crucial myelin-associated protein such as MBP, we performed Western blot analysis against MBP on our samples. The results in Figure 1C confirm that MBP is present in our isolated myelin debris fraction.

### 3.2. Myelin Debris Loading in Mouse and Human Microglia Activates Cholesteryl Ester (CE) Synthesis in Intact Cells

**Figure 2:**
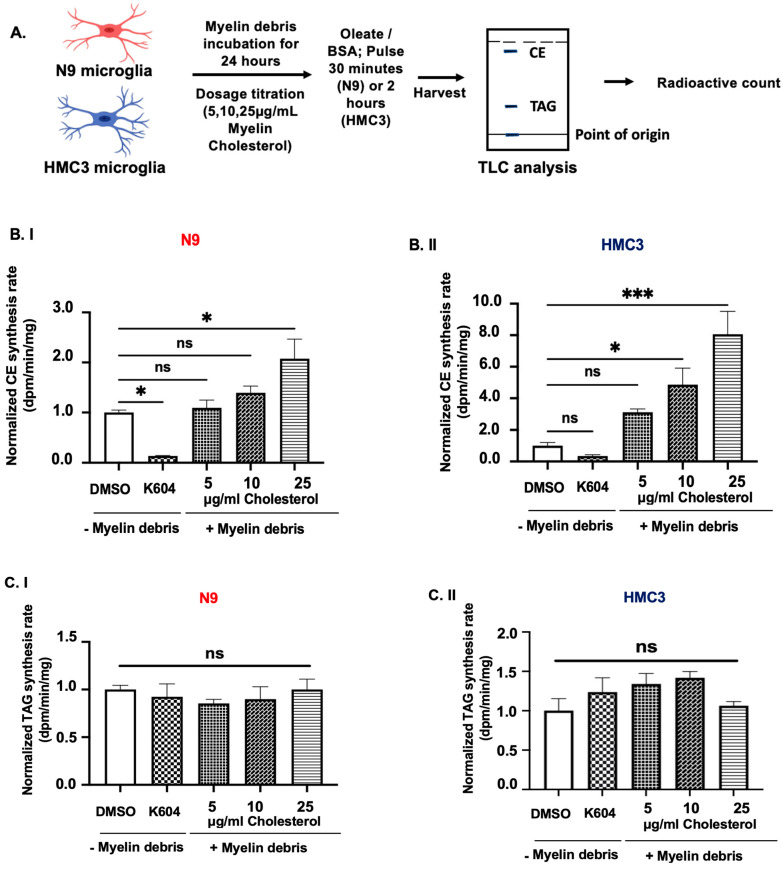
Myelin debris treatment in mouse and human microglia activates the synthesis of cholesteryl esters (CE), but not triacylglycerides (TAG), in a dose-dependent manner using oleic acid as substrate. (**A**) Treatment scheme: The extracted lipids underwent thin-layer chromatography (TLC) to separate individual lipids, which were identified by internal lipid standards (CEs and TAG) added during lipid extraction and as described in Section 2. Images were creared using BioRender. (**B**) CE and (**C**) TAG synthesis rates in intact (**I**) N9 cells and (**II**) HMC3 cells were monitored using a ^3^H-oleate pulse. Cells were grown to 80% confluency and treated with DMSO alone, with the ACAT1/SOAT1 inhibitor K604, or with myelin debris at 5, 10 and 25 μg/mL cholesterol for 24 h. Afterwards, CE and TAG biosynthesis rates in intact cells were monitored by providing ^3^H-oleate to intact N9 cells for 30 min and to HMC3 cells for 2 h. Cholesterol content in myelin debris was measured using the Cholesterol Wako kit. Raw radioactive counts were normalized to 119.8 dpm/min/mg (CE) and 2088.08 dpm/min/mg (TAG) for N9 cells, and 68.78 dpm/min/mg (CE) and 3933.08 dpm/min/mg (TAG) for HMC3 cells. N = 3. Data are expressed as mean ± SEM. * *p* < 0.05; *** *p* < 0.001.

We next investigated whether myelin debris loading alters CE synthesis by incubating two microglial cell lines with ^3^H-oleate for a short time period (described in Section 2). This method is used to measure the biosynthesis of CEs and TAGs (in addition to other lipids derived from ^3^H-oleyl coenzyme A) in intact cells. ^3^H-oleate uptake rate in cells is cell-line dependent, thus we exposed N9 cells and HMC3 cells to ^3^H-oleate with different incubation time to establish a comparable incorporation baseline between the two cell lines, as well as ensuring that a raw radioactive count is within the linear detection range [34]. Raw radioactive counts for each lipid species and cell lines exposed to DMSO control treatment are reported in the legend for Figure 2. For CE, the raw radioactive count for N9 cells was 119.8 dpm/min/mg and for HMC3 this was 68.78 dpm/min/mg. The equivalent values for TAG, were 2088.08 dpm/min/mg for N9 cells and 3933.08 dpm/min/mg for HMC3. We exposed N9 cells to mouse myelin debris and HMC3 cells to human myelin debris at various dosages for 24 h and monitored the biosynthesis of CE and TAG. We tested a range of 5–25 μg/mL of myelin cholesterol based on previous work by Nugent et al., 2020 [7]. The experimental parameters are graphically demonstrated in Figure 2A.

After their exposure to myelin debris, we monitored the rate of CE synthesis in intact cells as shown in Figure 2B.I,B.II. Our analysis revealed that, in N9 cells, the relative CE biosynthesis rate was upregulated by 9%, 39%, and 108% when exposed to 5, 10, and 25 μg/mL of myelin cholesterol, respectively. In HMC3 cells, the CE biosynthesis rate increased by 312%, 486%, and 806% at 5, 10, and 25 μg/mL of myelin cholesterol, respectively. These results indicate that, in response to myelin debris loading, both N9 mouse and human HMC3 microglial cells upregulate CE synthesis in intact cells. Our results show that the magnitude of CE synthesis in HMC3 cells in response to human myelin debris is significantly higher than in N9 cells.

We also monitored the rate of TAG biosynthesis in N9 and HMC3 intact cells using the ^3^H-oleate pulse method, as reported in Figure 2C.I,C.II. Our analysis showed that, unlike the CE biosynthesis rate, no statistically significant increase in TAG biosynthesis was observed under the same treatment conditions. However, the data suggest a trend towards a small, dose-dependent increase in TAG biosynthesis in N9 cells, but not in HMC3 cells.

### 3.3. Pharmaceutical Inhibition of ACAT1/SOAT1 Reduces CE Accumulation and Intracellular Cholesterol Content in HMC3 Treated Myelin Debris

**Figure 3:**
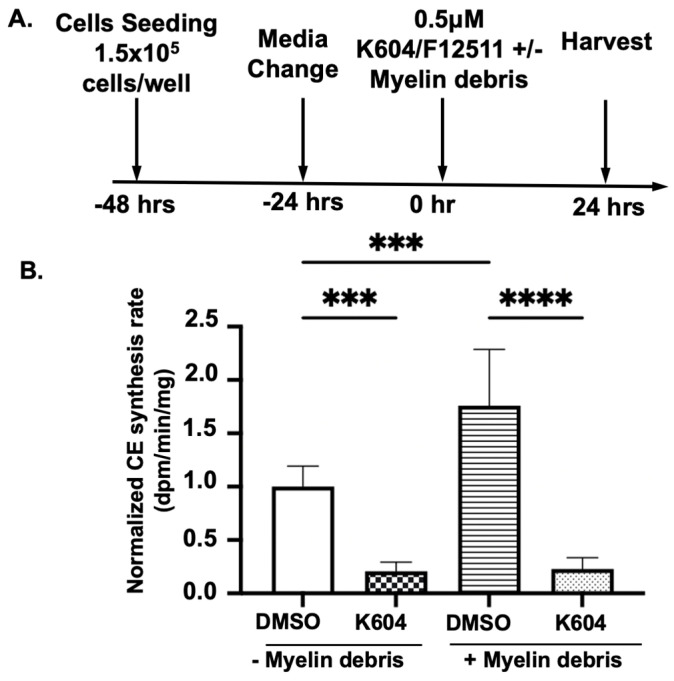

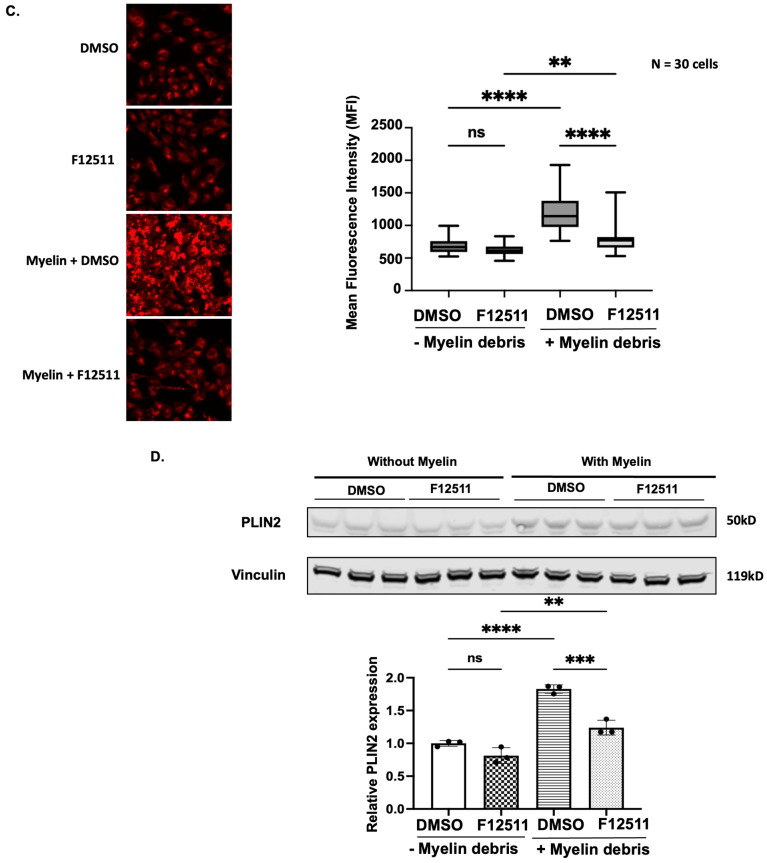

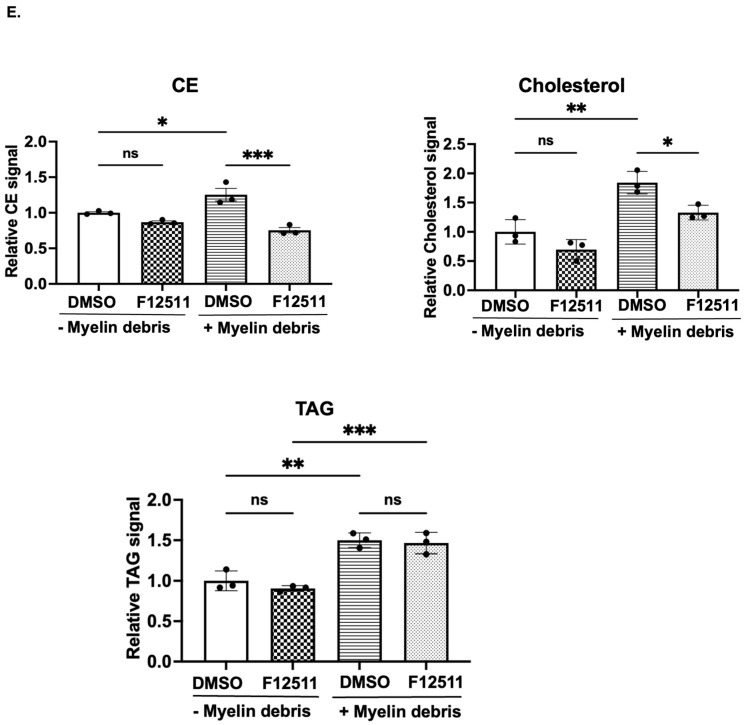
ACAT1 inhibition by K604 or F12511 reduces cholesteryl ester accumulation in HMC3 microglia treated with myelin debris. (**A**) Treatment scheme for all experiments. (**B**) HMC3 cells grown to 80% confluency were treated with DMSO, either with or without the ACAT1/SOAT1 inhibitor K604, or with myelin debris at 25 μg/mL cholesterol for 24 h as described in Section 2. Cholesteryl ester biosynthesis rates in intact cells were monitored by providing ^3^H-oleate to HMC3 for 2 h. Cholesterol content in myelin debris was measured using the Cholesterol Wako kit. Raw radioactive counts were normalized to 1, yielding 464.9 or 723.4 dpm/min/mg. (**C**) Representative image and quantification of neutral lipid staining by Nile Red staining, demonstrating lipid droplet accumulation in HMC3 cells. (**D**) Representative perilipin 2 (PLIN2) Western blot and quantification in HMC3 cells. (**E**) Quantification of TLC analysis for intracellular CE and free (unesterified cholesterol) and TAG in HMC3 cells. Values were calculated based on cells without myelin or ACAT1 inhibitors (K604 or F12511) treatment, normalized to 1. N = 3–6 for all experiments. Data are expressed as mean ± SEM. * *p* < 0.05; ** *p* < 0.01; *** *p* < 0.001; **** *p <* 0.0001. Western blot original images are in the Supplementary Materials.

Cholesterol from myelin debris can be converted to CE for storage by the cholesterol storage enzyme ACAT1/SOAT1 [53]. To test this possibility, we treated cells with or without myelin debris and the small molecule ACAT1 inhibitor K604 [47], followed by monitoring the rate of CE biosynthesis in intact HMC3 cells. The experimental scheme is illustrated in Figure 3A. The results in Figure 3B demonstrate that inhibition of ACAT1 in myelin debris-loaded HMC3 microglia reduced CE biosynthesis by approximately 87%.

To further validate these data, we used Nile Red, a commonly used neutral lipid stain for lipid droplets [54], to determine the effect of ACAT1 blockage in live HMC3 cells (Figure 3C). Nile Red stains both components of lipid droplets (CE and TAG) [55]. In cells with excess cholesterol loading, cholesterol is converted into CE in the form of lipid droplets, and, when the storage process is blocked by inactivating ACAT1, CE droplets disappear. These processes can be monitored quantitatively via Nile Red staining in living cells [43,44]. Here, we switched to use F12511 instead of K604 as the small molecule ACAT1 blocker for the following two reasons: Firstly, F12511 is a more potent ACAT1 inhibitor compared with K604 [43]. K604 has a K_i_ = 0.45 μmol/L, while F12511 has a K_i_ = 0.039 μmol/L for ACAT1 [46]. Secondly, F12511 has been widely studied [56,57,58,59,60], and (2) our laboratory recently developed a drug delivery system using F12511 to treat a mouse model for Alzheimer’s disease [59,60]. Thus, we moved forward with the use of F12511 in later experiments as these results can help future studies in vivo. As shown in Figure 3C, 24 h of myelin debris treatment increased the Nile Red signal in HMC3 cells by 71%, while ACAT1 inhibitor F12511 treatment largely reduced the Nile Red signal by 65%, These data agree with the ³H-oleate pulse data in the HMC3 cell line (Figure 2B.II and Figure 3B). We also found that, when comparing the cell population in the F12511-treated group (without myelin) to that in the F12511-treated group (with myelin), the latter population showed a higher level of Nile Red staining. (Figure 3C, comparing the second box to the fourth box). This result suggests that myelin debris-loaded HMC3 cells may increase other neutral lipids that are not CEs.

To validate the Nile Red staining data, we used Perilipin 2 (PLIN2), a protein marker of lipid droplets, to quantify lipid droplet levels in HMC3 cells under different treatment conditions. PLIN2 has been widely used by different laboratories as a marker to monitor lipid droplets [24,61,62]. As expected, PLIN2 Western blot data support our Nile Red staining data (Figure 3D): The PLIN2 signal in myelin debris-treated cells increased by 80% compared with DMSO-treated cells with no myelin debris (Figure 3D, third bar vs. first bar), and treatment with F12511 in myelin debris-treated cells reduced the PLIN2 signal by approximately 57% compared with myelin debris-treated cells alone (Figure 3D, fourth bar vs. third bar). Our results, shown in Figure 3C,D, are consistent with previously published findings that myelin debris treatment increases not only CEs but also other neutral lipids [7,24]. The identities of these non-CE neutral lipid species are largely unknown. As our ^3^H-oleate pulse results for both N9 and HMC3 cells (Figure 2C) show that myelin debris does not increase biosynthesis of oleate-derived TAG, it is possible that these non-CE, neutral lipid species may not use oleyl CoA as their precursor. In other words, myelin debris may increase the biosynthesis of certain neutral lipid species that do not contain oleate.

The results described above show that the HMC3 cell line is a good model by which to study cholesterol homeostasis in myelin debris-loaded microglia. We next assessed the effect of ACAT1 inhibition on intracellular cholesterol and cholesterol ester content in HMC3 microglia. We used thin-layer chromatography (TLC) to separate and quantify cellular free cholesterol (unesterified cholesterol), CE and TAG content, based on the method described in Macala et al. [45] and Harned et al. [34]. Our data (Figure 3E, top two panels) show that, while myelin debris increased both CE by 125% and free cholesterol content by 180% in HMC3 cells, interestingly, F12511 treatment not only reduced CE content by 50%, but also reduced free cholesterol content in myelin debris-treated cells by 51%. We believe this result is likely due to ACAT1 inhibition leading to an increase in cellular cholesterol efflux (to facilitate cellular cholesterol disposal). The action of ACAT inhibitors on cellular cholesterol efflux has been previously documented in many cell lines, but not in microglia [16,63].

The results in Figure 3E, left panel, show that a significant amount of CE is present in HMC3 cells without myelin debris treatment. Treating cells with F12511 for 24 h did not cause a large reduction in this “ACAT inhibitor insensitive” CE content. The origin of these CEs is unknown. We speculate that they might be derived from materials present in serum, that they may reside in the endolysosome compartment, and that their metabolic fate is not directly linked with ACAT1.

TAG is mainly synthesized by diglyceride acyltransferase-1 and-2 (DGAT1 and DGAT2). Prakash and Manchada et al., of Chopra’s lab, have recently reported that DGAT2 inhibitors could reduce lipid droplets in microglia induced by amyloid beta (Aβ) peptides [64]. However, their study did not use myelin debris-treated microglia and did not include use of ACAT inhibitors. Here, instead of testing the possible involvement of DGAT, we monitored the total TAG content upon myelin debris treatment with or without ACAT inhibitor and report the result in Figure 3E (bottom panel). The results show that myelin debris incubation did increase TAG content by 150%, and that treatment with F12511 did not impact the TAG level. Overall, these data are consistent with the data from the study of Nugent et al. in bone narrow-derived macrophages (BMDM) [7]. They have shown that both the diacylglycerides (DAG) and TAG contents are upregulated in BMDM, when incubated with myelin debris [7]. Myelin lipids contain a very high amount of saturated long chain fatty acids [65,66]. We speculate that, upon entering the cell interior, the saturated long-chain fatty acids become fatty acyl CoA to serve as a substrate for DGATs (or perhaps other enzymes) to produce TAG [40]; this process may not be easily detectable using a ^3^H-oleate pulse. Further investigations are needed to investigate the myelin debris–TAG connection.

### 3.4. Treatments with Myelin Debris and/or ACAT1 Inhibitor F12511 Do Not Change ACAT1 Protein Expression in HMC3 Cells

**Figure 4:**
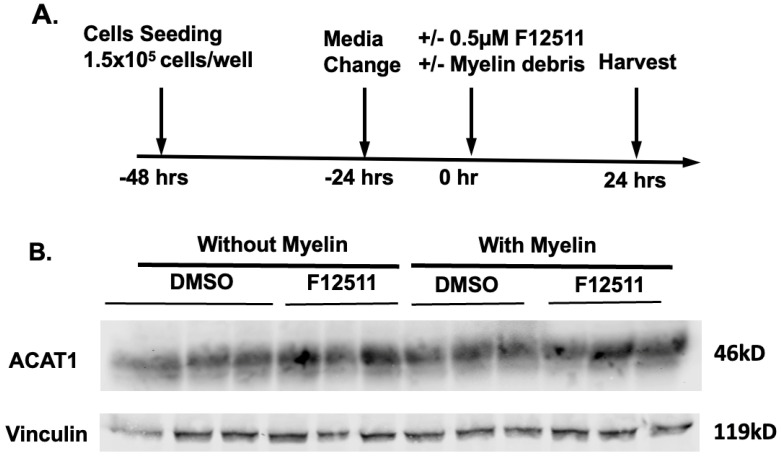

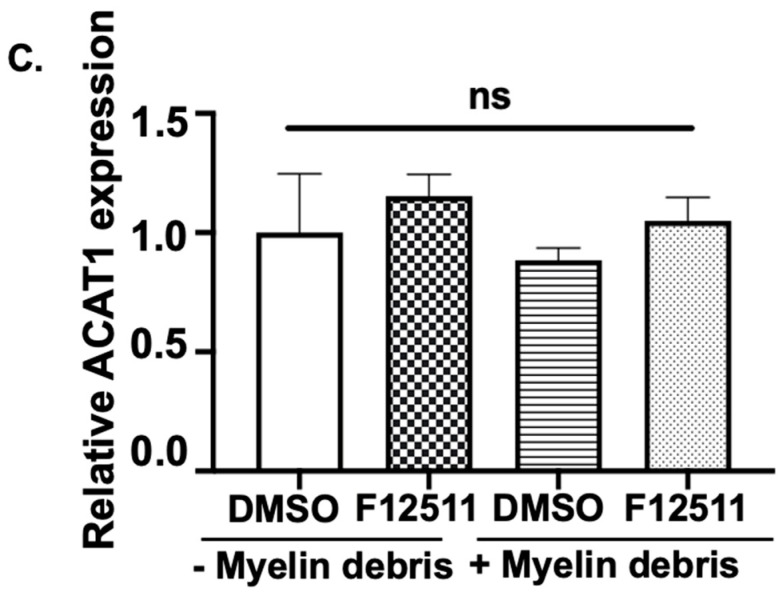
Treatment with myelin debris and/or the ACAT inhibitor K604 does not alter ACAT1 protein content in HMC3 cells. (**A**) Treatment scheme. HMC3 cells grown to 80% confluency were treated with DMSO, either with or without ACAT1 inhibitor F12511, or with myelin debris at 25 μg/mL cholesterol for 24 h. (**B**) Representative western blot. Cells were then harvested for Western blot analyses, with vinculin used as the loading control. (**C**) Quantification of western blot analysis. Values were calculated using the untreated cells (no myelin or K604) as 1. N = 3. Data are expressed as mean ± SEM. NS, not significant. Western blot original images are in the Supplementary Materials.

As myelin debris treatment increased CE synthesis and cellular CE content, while F12511 reduced these levels in HMC3 cells, we next asked whether these treatments affect ACAT1 protein expression. ACAT1 is a membrane-bound enzyme located in the endoplasmic reticulum (ER) and ER-associated membrane [34]. Previous work has shown that, in many cell types examined, the primary mode of ACAT1 regulation is attributed to allosteric regulation by its substrates, cholesterol, and oxysterols (as reviewed in [67]). F12511 is a potent inhibitor of ACAT1 [48], but F12511 may or may not alter ACAT1 protein expression. We analyzed whole HMC3 cell lysates treated with and without myelin debris, with and without F12511, with the experimental plan shown in Figure 4A, a representative Western blot shown in Figure 4B, and the quantitative analysis shown in Figure 4C. Our analysis demonstrated that ACAT1 protein content does not change under any experimental condition, with or without myelin debris, and with or without F12511. As myelin debris is cholesterol-rich, the increase in CE biosynthesis, as demonstrated in Figure 2B.I,B.II and Figure 3B, is likely due to the increased availability of cholesterol as substrate to ACAT1 in the ER when cells are exposed to myelin debris.

### 3.5. Pharmaceutical Inhibition of ACAT1 for 24 h in Myelin Debris-Loaded HMC3 Microglia Upregulates the Protein Expression of the Cholesterol Efflux Transporter ABCA1

**Figure 5:**
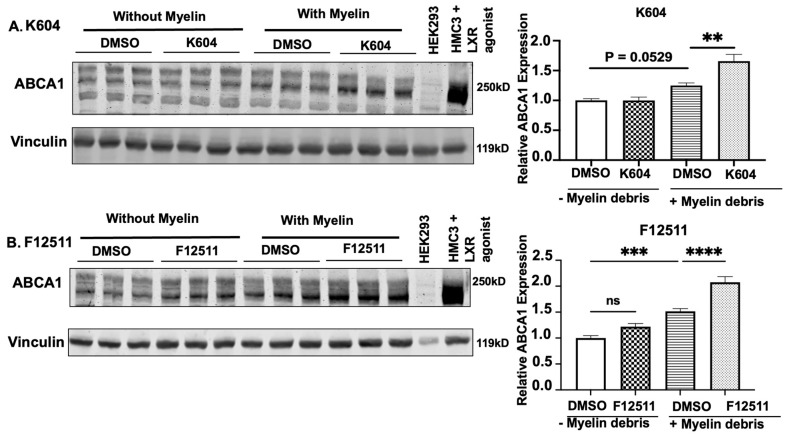
ABCA1 protein content increased after 24 h treatment with human myelin debris co-incubated with either K604 or F12511. Human microglial HMC3 cells grown to 80% confluency and treated with (**A**) 0.5μM K604 or (**B**) 0.5μM F12511, incubated with or without human myelin debris at 25 μg/mL cholesterol. Cells were then harvested for Western blot analyses. HEK293 cell lysate was used as a negative control for ABCA1, and HMC3 treated with 10 μM LXR agonist T0901317 for 24 h served as a positive control for ABCA1. Vinculin was the loading control. Values were calculated based on the untreated condition (no myelin and without K604 or F12511), normalized to 1. N = 3–6. Data are expressed as mean ± SEM. ** *p* < 0.01; *** *p* < 0.001; **** *p <* 0.0001. Western blot original images are in the Supplementary Materials.

Cholesterol efflux is an important step to re-establishing cholesterol homeostasis in microglia and restoring their proper function (reviewed in [28]). Removal of excess cholesterol in cells occurs through various ABC transporters, most prominently through ABCA1 [23]. We used two different ACAT1 inhibitors, K604 and F12511, to monitor ABCA1 protein content in the whole cell lysate of HMC3 cells loaded with myelin debris for 24 h. HEK-293 cells, which lack ABCA1, were used as a negative control [16]. ABCA1 is known to be highly upregulated by LXR agonists [68]. We used the LXR agonist T0901317 to serve as a positive control [35]. The results reported in Figure 5A,B indicate that total ABCA1 protein content is significantly upregulated with either K604 or F12511 when compared with cells treated with myelin debris alone. Quantitative analyses revealed approximately 32% and 36% increases in ABCA1 protein content in myelin debris-loaded cells treated with ACAT1 inhibitors K604 and F12511, respectively.

We also observed that, while myelin debris loading in HMC3 cells increased ABCA1 protein content by 25–50% (Figure 5A,B), adding ACAT1 inhibitors further increased ABCA1 protein content. These results, along with the findings shown in Figure 3E, suggest that ACAT1 inhibitors enhanced cholesterol efflux activity via ABCA1 upregulation, resulting in a decrease in the intracellular cholesterol pool. We conclude that, in myelin debris-loaded microglia, ACAT1 inhibitors not only ameliorate CE accumulation but also enhance cellular cholesterol disposal through the upregulation of the ABCA1 protein content.

Previously, we had reported that, in mouse microglia N9 cells grown in medium-containing serum, adding K604 for up to 8 h had no detectable effect on cellular ABCA1 protein levels [32]. A comparison of our current results (Figure 5A,B) with previous results suggests that, in microglia, the effect of the ACAT inhibitor is dependent on cellular cholesterol loading. The magnitude of this effect is greater and easier to detect when cells are loaded with cholesterol-rich substances such as myelin debris.

### 3.6. F12511 Treatment in Myelin Debris-Loaded HMC3 Microglia Significantly Increases ABCA1 mRNA Expression

**Figure 6:**
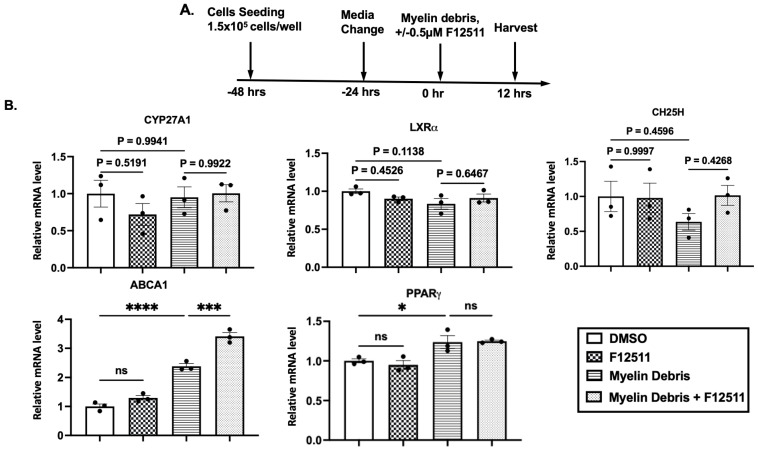
F12511 treatment in myelin debris-loaded HMC3 microglia significantly increases *ABCA1* mRNA expression. Myelin debris also activates *PPARγ* gene expression in HMC3 microglia. (**A**) Treatment scheme. (**B**) Treated HMC3 cells were lysed in Trizol, and RNA was purified. RNA samples were then analyzed using NanoString Elements. Bar graphs display the relative mRNA expression for key genes (*ABCA1*, *PPARγ*, *CH25H*, *CYP27A1* and *LXRa*) after normalization to the housekeeping gene (*ABCF1*). Gene expression in DMSO-treated cells (without myelin) was normalized to 1. N = 3. Data are expressed as mean ± SEM. * *p* < 0.05; *** *p* < 0.001; and **** *p* < 0.0001. Primers sequence are reported in Table 1.

When ACAT1 is blocked, excess cholesterol released from the ACAT1 storage pool in the ER can participate in cholesterol trafficking to other membrane compartments [69]. This excess cholesterol can either convert to oxysterols to activate LXR, the master regulator that induces ABCA1 gene expression [68,70] or move to the plasma membrane to stabilize ABCA1 protein and decrease its turnover rate, thereby increasing cellular ABCA1 protein content [71]. Both events may occur. To determine whether ACAT1 inhibitor affects *ABCA1* gene expression, we treated HMC3 cells with or without myelin debris and with or without F12511 for 12 h, followed by RNA extraction and analysis using NanoString Elements technology (Figure 6A). NanoString Elements enables the quantification of a wide range of genes in a single reaction via hybridization and individual barcoding, without the amplification required by traditional qPCR.

In addition to *ABCA1* gene expression, we also examined other related genes involved in intracellular cholesterol and lipid processing pathways that are known to be upstream of *ABCA1*, including peroxisome proliferator-activated receptor gamma (*PPARγ*) (lipid processing), liver X receptor alpha (*LXRα*) (a direct upstream regulator of ABCA1), cholesterol 25-hydroxylase (*CH25H*), and sterol 27-hydroxylase (*CYP27A1*) (oxysterol-converting enzymes). The data collected from this experiment were quantified and plotted in Figure 6B.

The results show that, firstly, and in agreement with our protein content analysis data (Figure 5), *ABCA1* gene expression is significantly upregulated in myelin debris-loaded HMC3 microglia by about 80% and is further increased by another 43% in cells treated with both myelin debris and F12511 (Figure 6B). This result suggests that ACAT1 inhibition can upregulate *ABCA1* gene expression in myelin debris-treated cells.

Second, we observed an increase in *PPARγ* gene expression when cells were exposed to myelin debris. This result is consistent with previously published data in macrophages from a multiple sclerosis model [72] (Figure 6B). Most likely, phosphatidylserine (PS), a component of myelin debris, is responsible for the upregulation of *PPARγ* gene expression [72]. Treatment with F12511 did not alter *PPARγ* expression in myelin debris-treated cells (Figure 6B).

Third, regarding the two oxysterol-converting enzymes (CH25H and CYP27A1), we did not observe any significant changes across all treatment conditions, nor did we see changes in the direct upstream regulator of *ABCA1* gene expression (*LXRα*). We speculate that, in order to observe upregulation of *LXRα* and other oxysterol-converting enzyme gene expression, we may need to analyze treated cells at earlier time points, such as 4, 6, and 8 h. It is also possible that the activation of these genes is transient, and that they returned to normal levels by 12 h, while *ABCA1* gene expression remained upregulated at 12 h to induce ABCA1 protein expression for efflux at 24 h (Figure 5 and Figure 6B).

### 3.7. Liver X Receptors (LXR) Antagonist GSK2033 Treatment Blocks ABCA1 Protein Expression in Myelin Debris and ACAT1 Inhibitor-Treated HMC3 Microglial Cells

**Figure 7:**
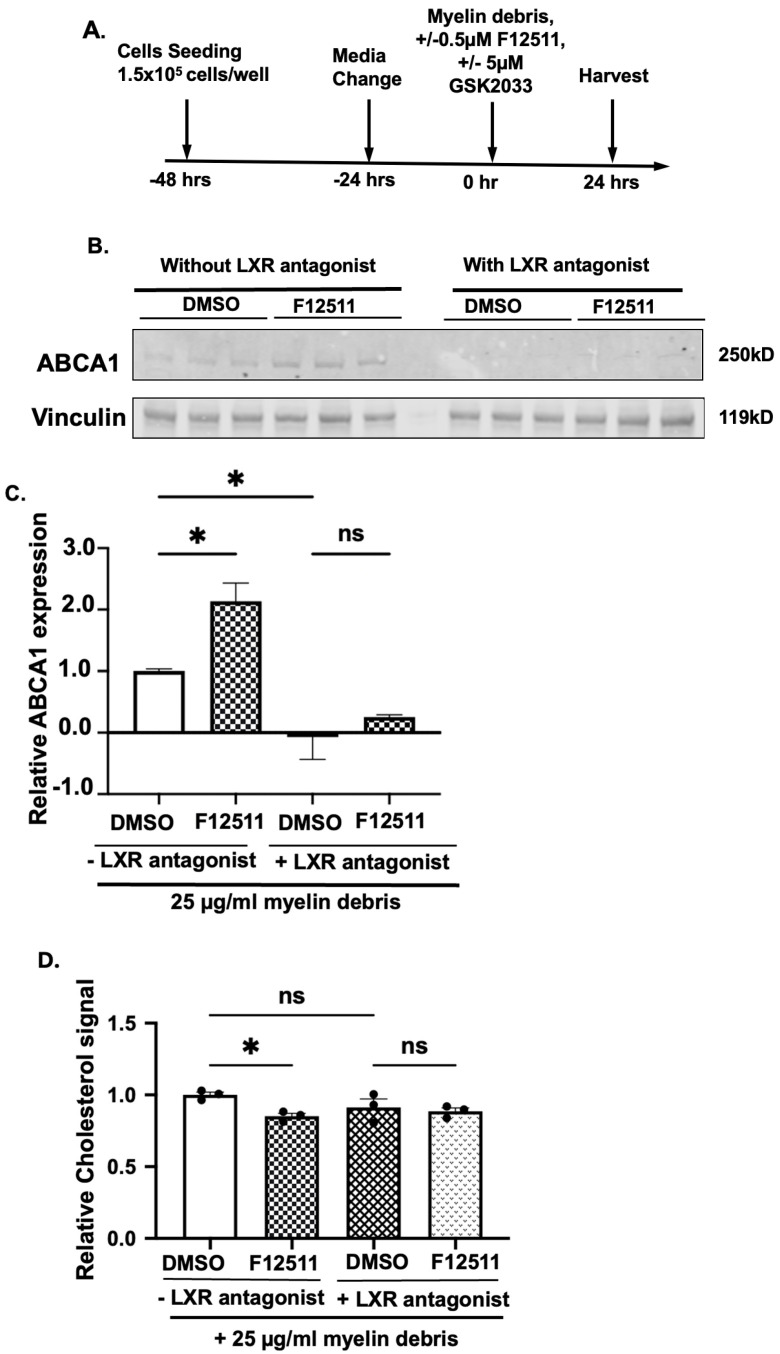
LXR antagonist GSK2033 treatment blocks ABCA1 protein expression in myelin debris and ACAT1 inhibitor-treated HMC3 microglial cells. (**A**) Treatment scheme. (**B**) Representative Western blot. (**C**) Quantification of the Western blot. Vinculin was used as the loading control. Values were calculated based on the myelin-treated cells without LXR agonist and F12511, normalized to 1. (**D**) Quantification of TLC analysis for intracellular free unesterified cholesterol in HMC3 cells. Values were calculated based on cells without LXR antagonist GSK2033 treatment, normalized to 1. N = 3 for all experiments. Data are expressed as mean ± SEM. * *p* < 0.05. Western blot original images are in the Supplementary Materials.

As we were unable to observe *LXRα* gene upregulation at the 12 h treatment time point, we took an alternative approach to validate that the effect of the ACAT1 inhibitor on ABCA1 expression is LXR-dependent. We employed a specific LXR antagonist, GSK2033 [36,73], in our system (Figure 7A). Briefly, the LXR antagonist was added simultaneously with F12511 to these cells, and we monitored ABCA1 protein expression via Western blot (Figure 7A,B). As reported in Figure 7C, quantitative analysis of the Western blot results demonstrated that GSK2033 effectively diminished ABCA1 protein expression in both DMSO and F12511-treated cells with myelin debris, supporting our conclusion that the effect of F12511 on ABCA1 protein expression is LXR-dependent. To confirm whether diminishing ABCA1 content in myelin debris-loaded microglia treated with F12511 also abolishes its ability to reduce intracellular cholesterol, we preincubated cells with myelin debris and treated them with or without F12511 or with or without GSK2033 and followed this with TLC analysis. Our data in Figure 7D suggest that GSK2033 blocked F12511 action on intracellular cholesterol efflux, indicating that F12511 action in myelin debris-loaded HMC3 microglia is dependent on LXR.

## 4. Discussion

Alzheimer’s disease (AD) is classified as early onset (EOAD) and late onset (LOAD), with LOAD (affecting patients aged 65 or older) comprising more than 90% of all AD cases [74]. While mouse models for LOAD are becoming available [11], no simple cell culture model for LOAD is currently available. Myelin debris-loaded microglia have been considered as a cell model for aging. In our current work, we used two established microglia cell lines, mouse N9 and human HMC3, to gain a better understanding of how myelin debris-loaded microglia respond to the pharmaceutical inhibition of ACAT1.

**Figure 8:**
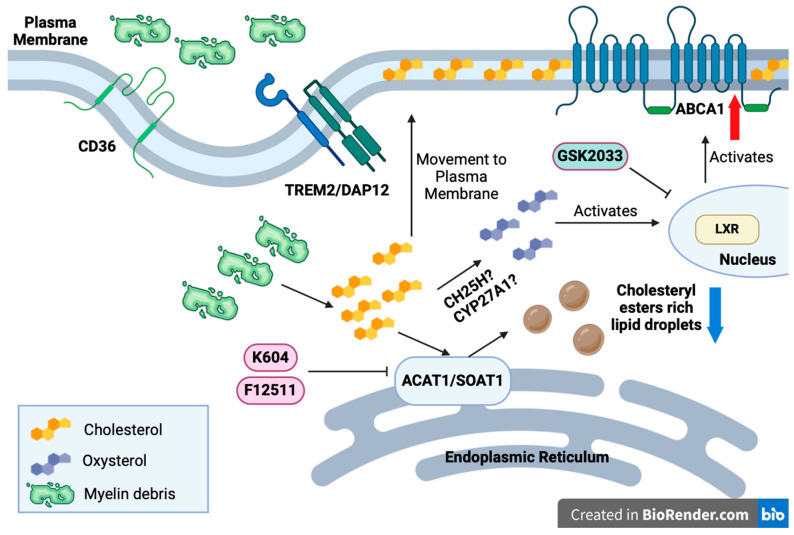
A working model illustrating the mechanism of ACAT1/SOAT1 inhibition in HMC3 microglia. With aging, myelin debris accumulates and is phagocytosed by microglia via receptors such as CD36, TREM2/DAP12, etc. Myelin debris is enriched in cholesterol, which can be stored as cholesteryl esters droplets by ACAT1/SOAT1 at the ER. This leads to CE lipid droplet accumulation in aging microglia. Pharmaceutical inhibition of ACAT1/SOAT1 by K604 or F12511 releases an extra pool of cholesterol to participate in cholesterol turnover. The cholesterol can then (1) migrate to the lipid–raft regions in the plasma membrane to serve as a substrate for the major lipid efflux protein and participate in cholesterol efflux, or (2) be converted into oxysterols by CH25OH, or other enzymes such as CYP27A1, which activates LXR and leads to the upregulation of ABCA1 expression for cholesterol efflux. Additionally, oxysterols can cross the blood–brain barrier into the periphery, restoring cholesterol homeostasis in the microglia and the brain.

Using ^3^H-Oleate pulse-chase assay, we demonstrated that in both N9 and HMC3 cells, CE biosynthesis increases in a myelin debris dose-dependent manner (Figure 2). We then showed that, in myelin debris-loaded HMC3 microglia, inhibition of ACAT1 alleviates CE and cholesterol accumulation (Figure 3) without impacting the ACAT1 protein expression (Figure 4). We monitored the downstream effects of ACAT1/SOAT1 inhibition and found that treatment with two different ACAT1 inhibitors, K604 and F12511, upregulated ABCA1 protein expression (Figure 5) and ABCA1 gene expression (Figure 6). These results correlate with a decrease in cellular cholesterol content (Figure 3), suggesting that increased cholesterol efflux occurs in myelin debris-loaded HMC3 microglia upon ACAT1 inhibition. We then showed that the effect of the ACAT1 inhibitor on ABCA1 expression and on cellular cholesterol content are most likely LXR-dependent (Figure 7). We built a working model (illustrated in Figure 8) to explain the action of ACAT1 inhibition in myelin debris-loaded microglia. This model is elaborated in more detail below.

As illustrated in Figure 8, myelin debris enters the cell surface of microglia via phagocytic receptors and unloads its cholesterol to the ER, where ACAT1 is localized. ACAT1 converts cholesterol unloaded from myelin debris into CEs for storage. Blocking ACAT1 activity diverts the cholesterol storage pool, to participate in the cholesterol translocation and turnover process. The diverted cholesterol pool can (1) migrate to the plasma membrane to serve as a substrate for efflux or (2) be converted into oxysterols, such as 27-OH cholesterol, 25-OH oxysterols, etc. Oxysterols can serve as ligands to activate LXR, which then upregulates *ABCA1* expression. ABCA1 protein is one of the most important proteins involved in the cellular cholesterol efflux process and has been linked to beneficial responses, including producing anti-inflammatory effects in various diseases (as reviewed in [75,76,77,78]). In macrophages, ABCA1 functions as an anti-inflammatory receptor [79].

The scheme described above provides a rationale by which to explain why blocking ACAT1 can restore cholesterol homeostasis in microglia. There are at least two possible mechanisms to explain ABCA1 upregulation by ACAT1 inhibition: (1) an increase in cholesterol availability at the plasma membrane as a substrate for ABCA1, and/or (2) an increase in oxysterols, perhaps by providing more cholesterol as a substrate to the enzymes such as CH25H, CYP27A1, or other oxysterol-converting enzymes. The increase in oxysterol concentration then activates the LXR pathway, which upregulates the ABCA1 protein and *ABCA1* mRNA expression [16,80].

Upregulation of ABCA1 can also be achieved through the use of synthetic LXR agonists. However, the difficulty of using synthetic LXR agonists is that they are known to also stimulate the gene expression of SREBP1-c, which overproduces fatty acids and leads to an increase in TG biosynthesis. LXR agonist is expected to lead to hypertriglyceridemia [81]. Here, we show that feeding myelin debris-treated microglia with the potent ACAT1 inhibitor F12511 inhibits CE synthesis and induces the ABCA1 gene expression. As a result, F12511 led to reductions in cellular CE and cholesterol contents, without increasing the TAG content in microglia. These results suggest that using ACAT1 inhibitor is an alternative to using synthetic LXR agonists to increase ABCA1 protein expression.

## 5. Conclusions

In this work, we demonstrated that ACAT1 inhibition activates ABCA1 gene expression in a LXR dependent manner in myelin debris loaded microglia. ACAT1 inhibition upregulates cholesterol efflux in cholesterol burden cells through ABCA1 activation. Our study highlights the role of ACAT1 as a promising therapeutic target for treating AD.

Besides aging, apolipoprotein E4 (*ApoE4*) is the biggest genetic risk factor for developing AD and has been linked to disruption in cholesterol homeostasis [22,62,82,83]. APOE4 expression in microglia-phagocytosed myelin debris leads to the accumulation of neutral lipids and lysosomal mass [61]. Additionally, APOE4 expression leads to aberrant cholesterol trafficking and perturbed cholesterol metabolism across various different cell types in the brain [22,62,82,83]. In APOE4 postmortem human brain tissue, CE is upregulated [82]. It remains unknown if ACAT1 inhibition would be beneficial in an aging APOE4 model, so this should be carefully investigated.

For future studies, these proposed mechanisms (Figure 8) need to be elucidated in microglia at the biochemical and cell biological levels. Additionally, oxysterols can be cleared from the brain into the periphery, which has been proposed as one of the main mechanisms for cholesterol disposal in the brain [84]. Previously, *ACAT1* genetic ablation and pharmaceutical inhibition have been reported to provide beneficial effects in various AD mouse models [17,59,85,86,87,88]. Our current work, along with the work of Nugent et al. [7], suggests that the pharmaceutical inhibition of ACAT1 may be a novel strategy by which to remove excess CE and cholesterol from the brain and attenuate the pro-inflammatory response in the brain. This possibility needs to be tested at the in vivo level.

## Figures and Tables

**Table 1:** Primer sequence.

Gene	Probe A (5′-3′)	Probe B (5′-3′)
ABCA1	GGTCTGAGAGCCGGTCATCAATCTCATGAAAGAGTTCCACAAAGGCTCCACAATTCTGCGGGTTAGCAGGAAGGTTAGGGAAC	CGAAAGCCATGACCTCCGATCACTCGAATATTTCTTCCAGGGTCGTCTCTGAGATGCCATAACTAGAAATGCCCA
PPARγ	GTACTCTTGAAGTTTCAGGTCATACTTGTAATCTGCAACCACTGGATCTGCATCCTCTTCTTTTCTTGGTGTTGAGAAGATGCTC	CGAAAGCCATGACCTCCGATCACTCTTCTCAGAATAATAAGGTGGAGATGCAGGCTCCACTTTGATTGCACTTTG
CH25H	ATGTCGAAGAAGCCCAAAGAAAACAGTTCCCAGACGCTCATATACTGCGTCAAAGACGCCTATCTTCCAGTTTGATCGGGAAACT	CGAAAGCCATGACCTCCGATCACTCTGAGCGGGTGGCACCCGAGCAGTGTGACGTTCATC
CYP27A1	GATGGATCGCTGCAGGCAGCCAATGCGTTTCTCGAACAGGATGTAGCAAACTGTTGAGATTATTGAGCTTCATCATGACCAGAAG	CGAAAGCCATGACCTCCGATCACTCTTCTGGAACATTAACCCGATGGATCTGACGAAGGTCACGGTGTCCTCGGG
LXRα	AGGCAGCCACCAGGCCTCAGCCATCCGGCCAAGAAAACAGAAAATATGGGCCTCAAGACCTAAGCGACAGCGTGACCTTGTTTCA	CGAAAGCCATGACCTCCGATCACTCAGGAATGTTTGCCCTTCTCAGTCTGTTCCACTTCTAGG
ABCF1 (housekeeping)	CCAGCTTGATGTCAGATGCATTTTCTAACATGGCTTGGCGGGAGGACATCCTTTCGGGTTATATCTATCATTTACTTGACACCCT	CGAAAGCCATGACCTCCGATCACTCGTCTGCATTGACGAACAGCTCCTTGCCATGAGCGGAGATGCTGAACTTCT

## Data Availability

The data presented in this study are available in this article.

## Supplementary Materials

The following supporting information can be downloaded at: https://www.mdpi.com/article/10.3390/biom14101301/s1.
