# Supplementary material for: Inhibiting the Cholesterol Storage Enzyme ACAT1/SOAT1 in Myelin Debris-Treated Microglial Cell Lines Activates the Gene Expression of Cholesterol Efflux Transporter ABCA1
Source: Biomolecules. 2024 Oct 14;14(10):1301. doi: 10.3390/biom14101301 (PMC11505751; doi:10.3390/biom14101301)

Figure S1. Figure 1C.

Mouse myelin

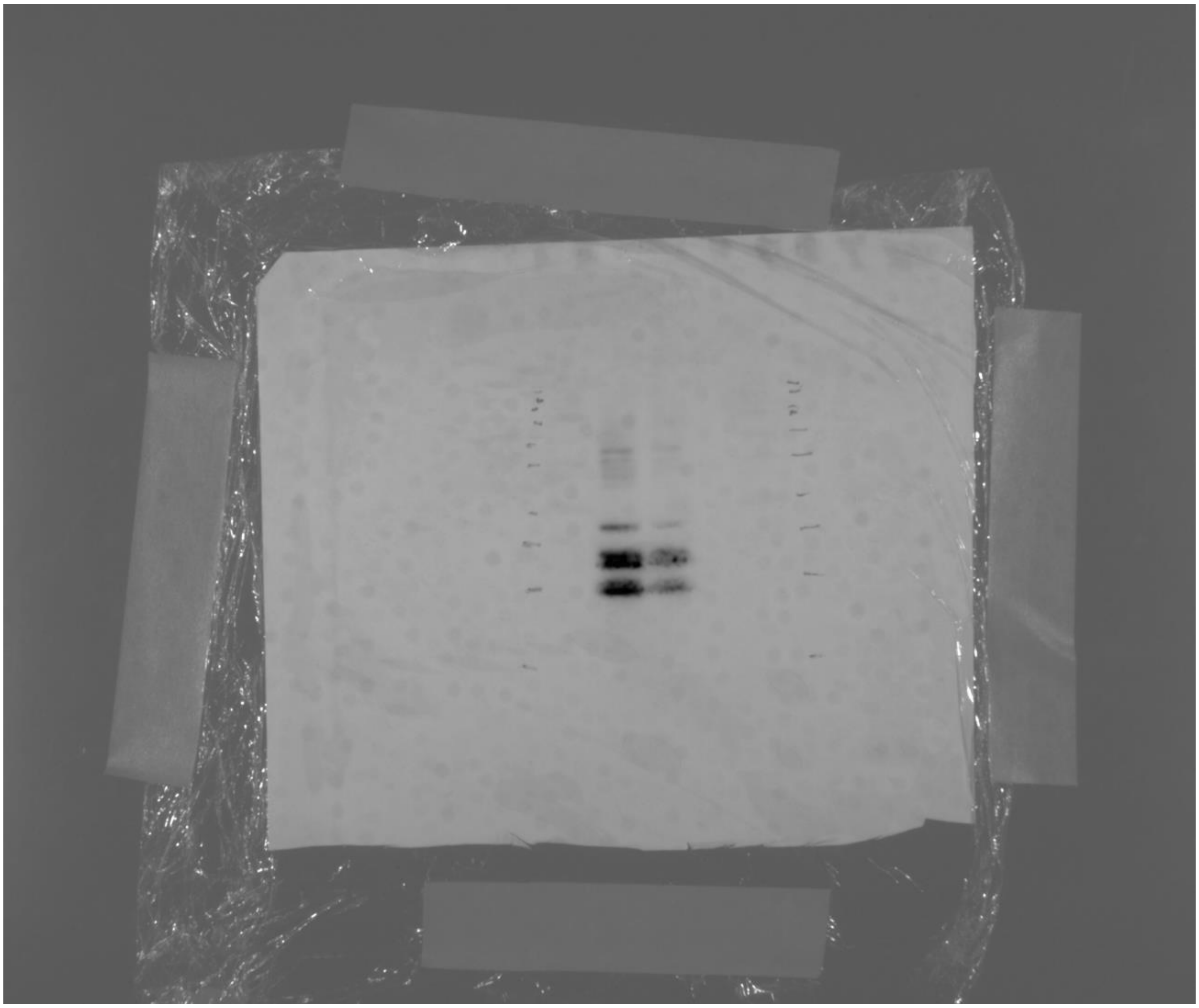

Figure S2. Figure 1C

Human Myelin

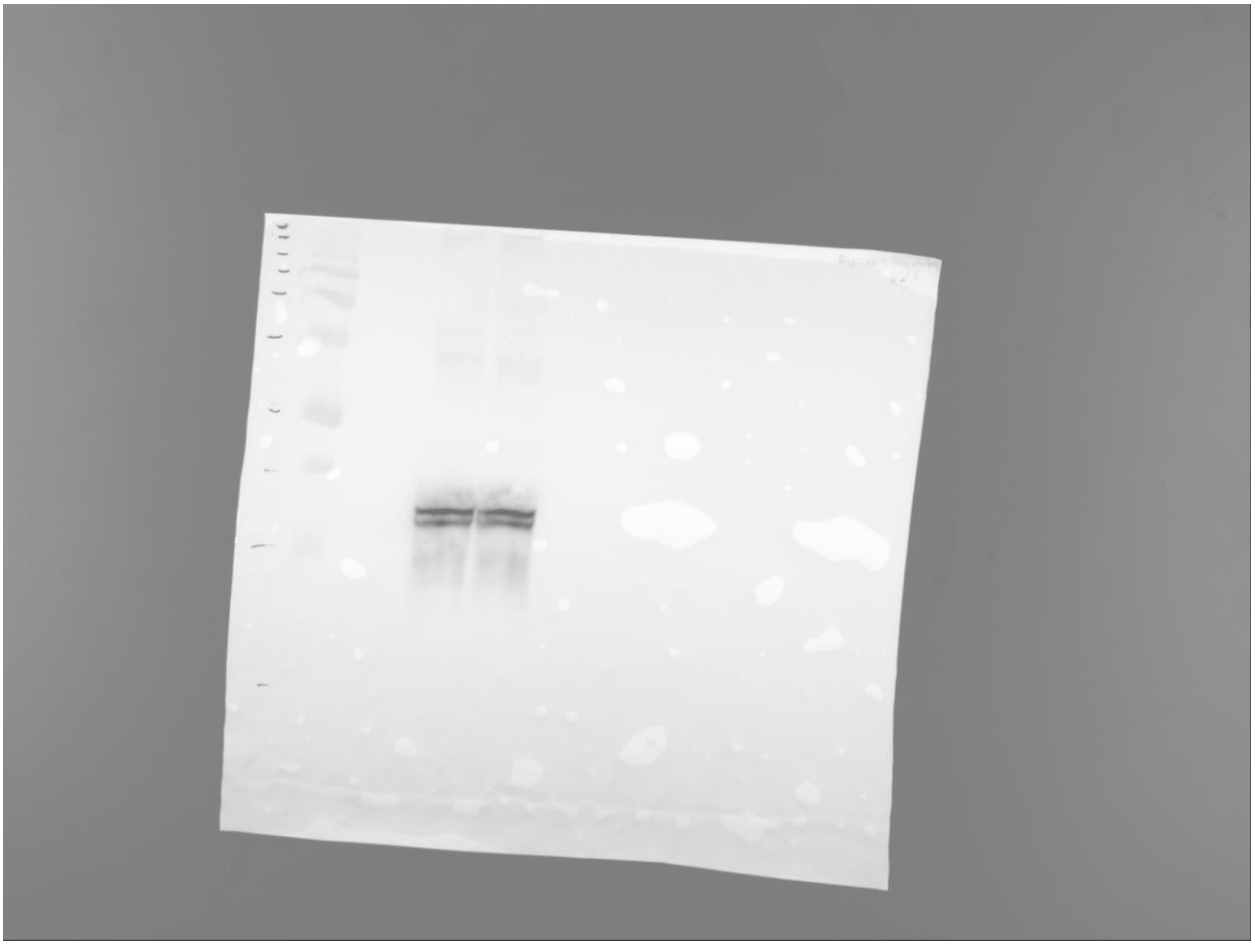

Figure S3. Figure 3D

PLIN2 (rabbit channel)

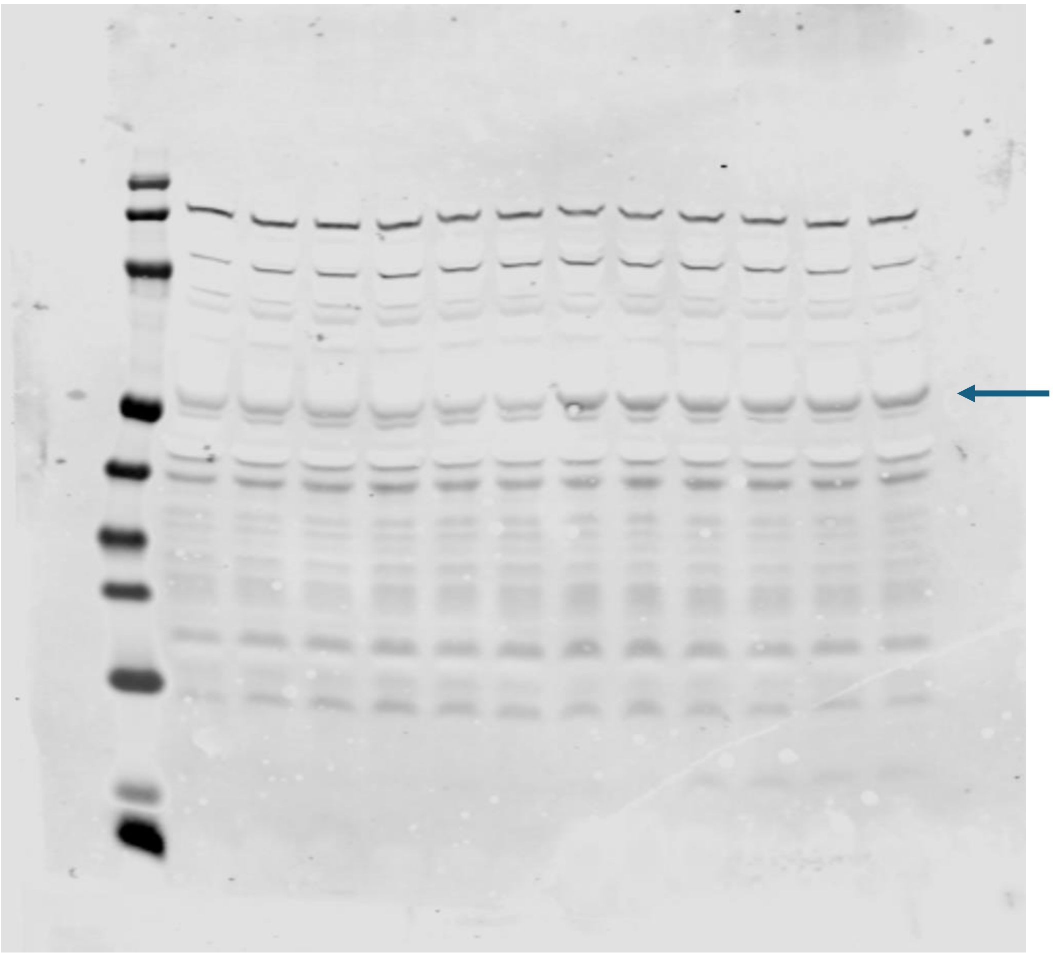

Vinculin (mouse channel)

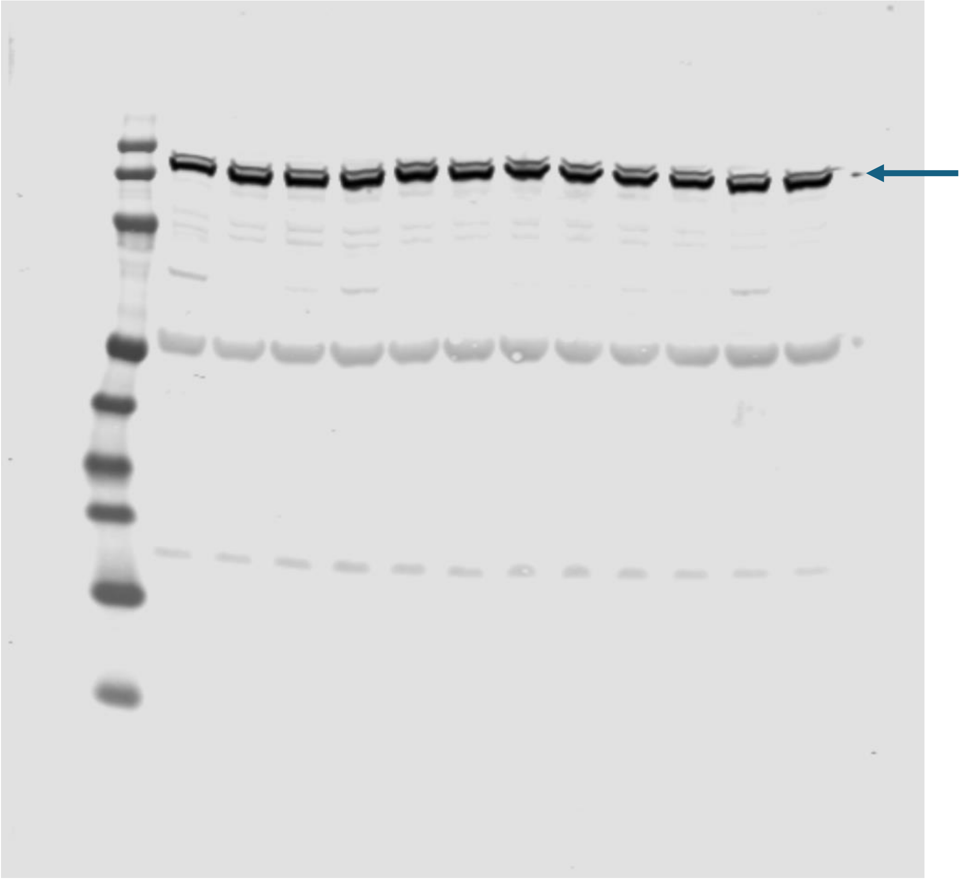

Dual channel

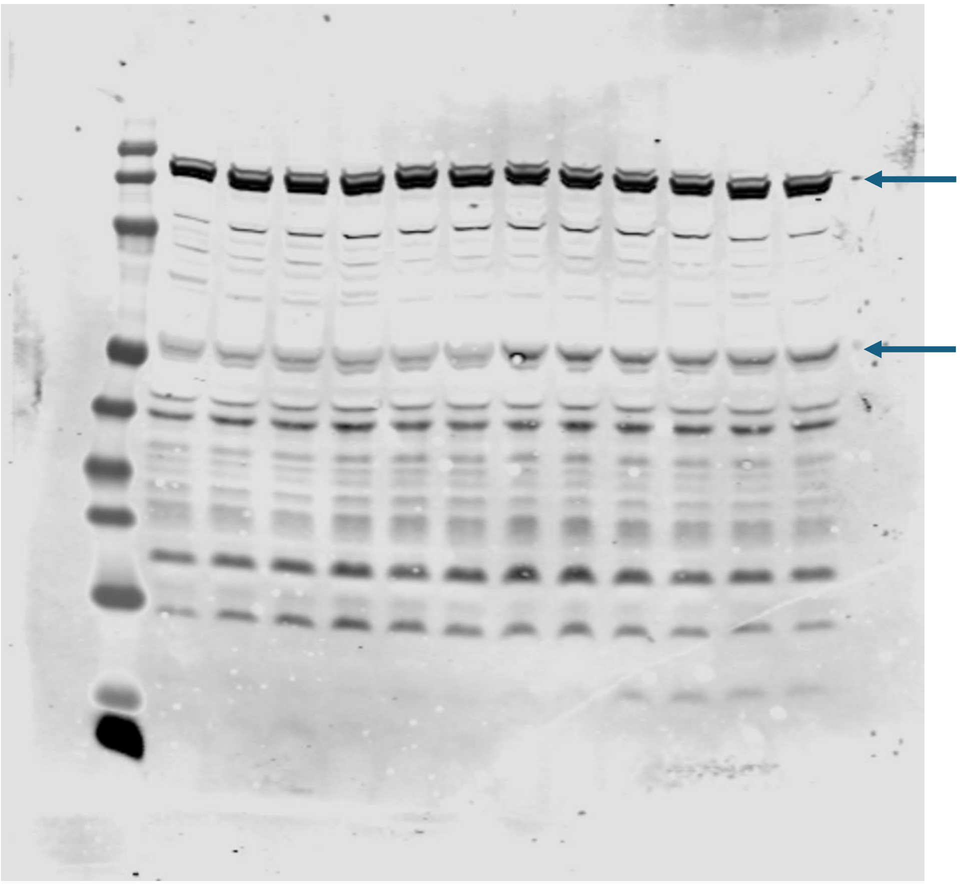

Figure S4. Figure 4B

ACAT1 (rabbit channel)

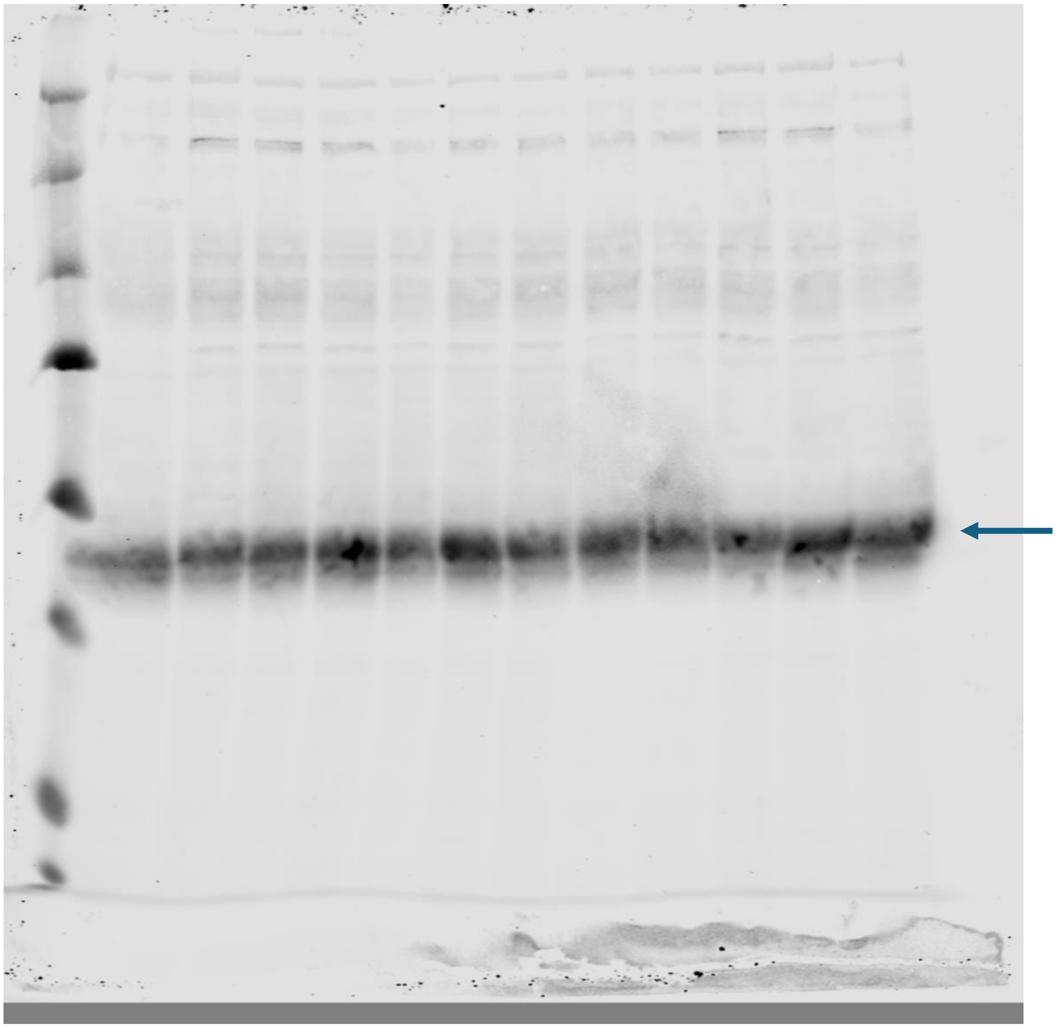

Vinculin (mouse channel)

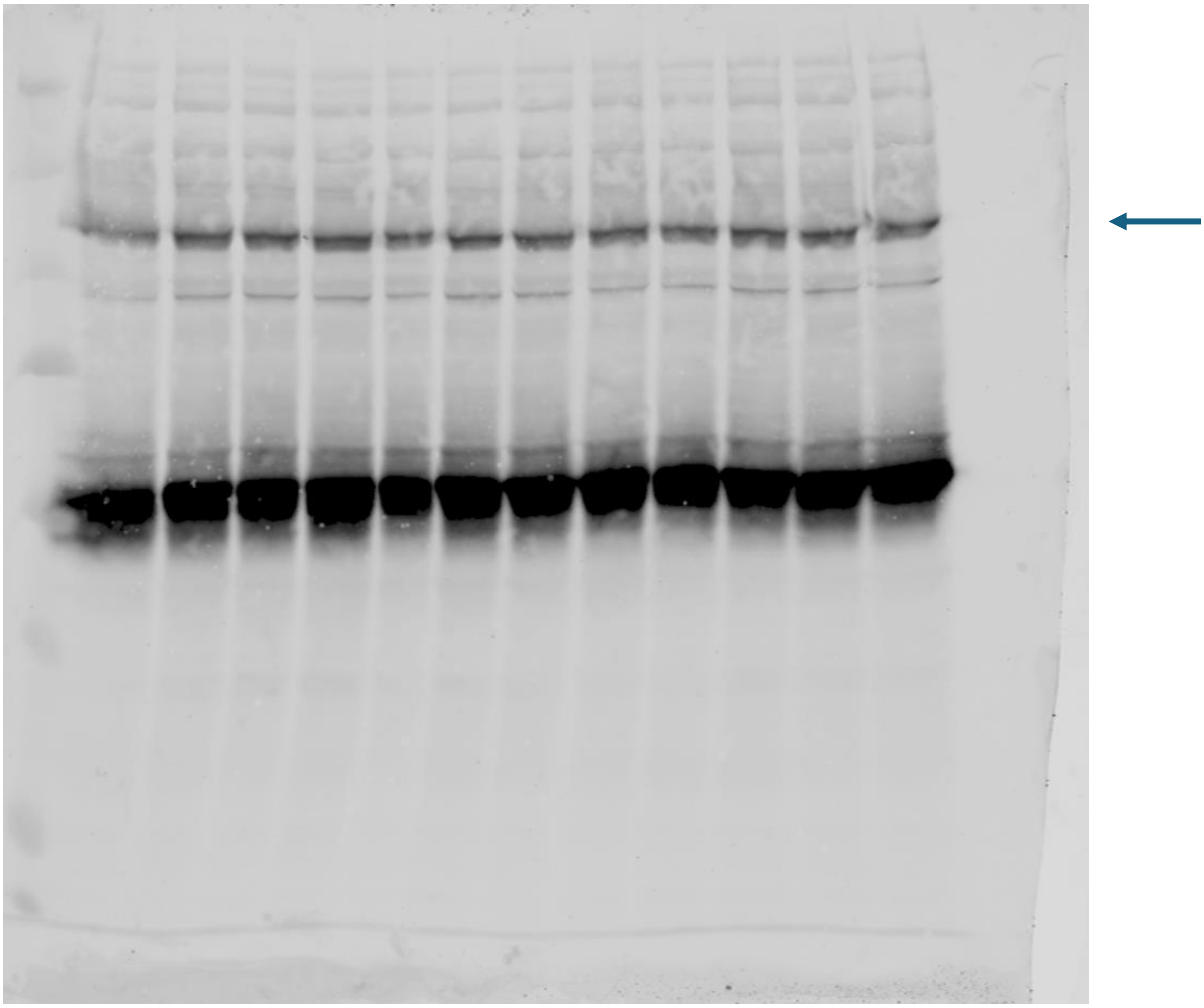

Dual channel

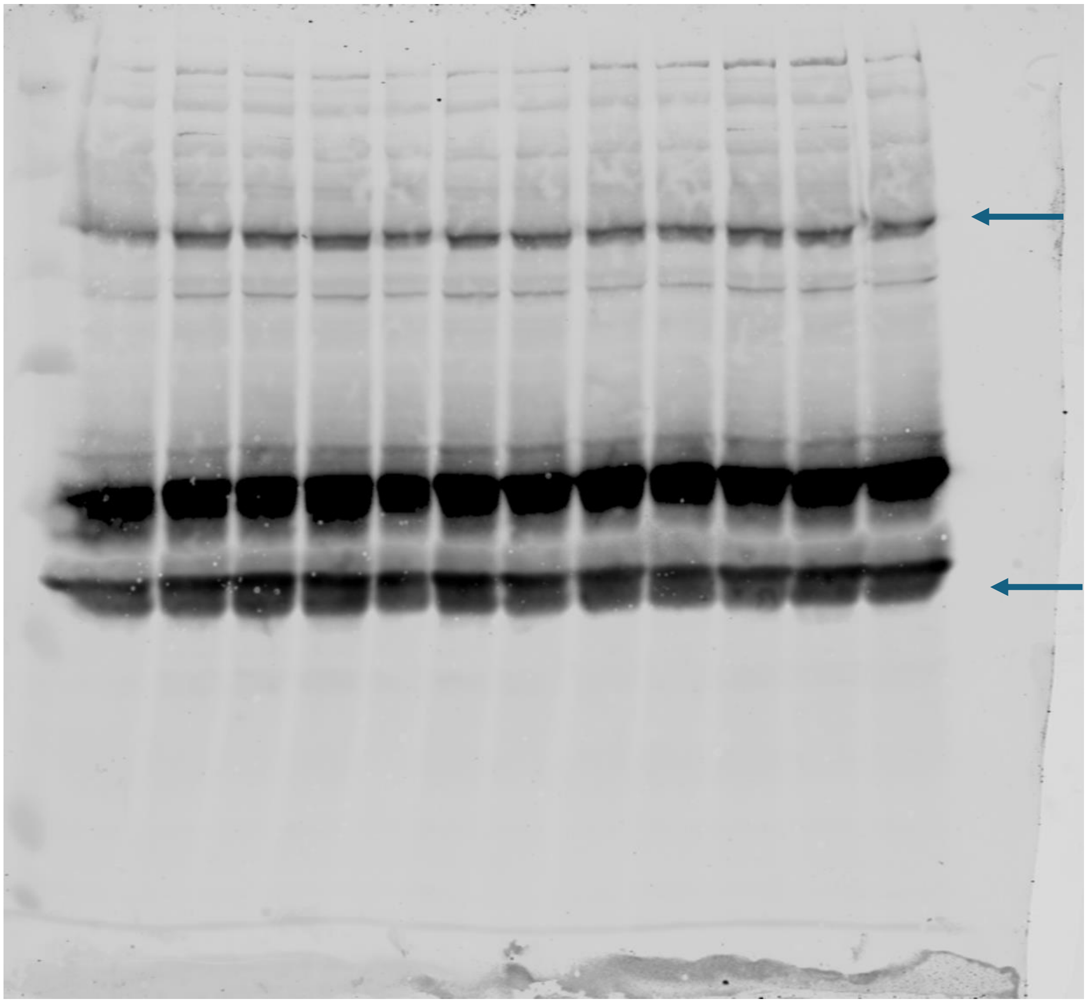

Figure S5. Figure 5A

ABCA1 (rabbit)

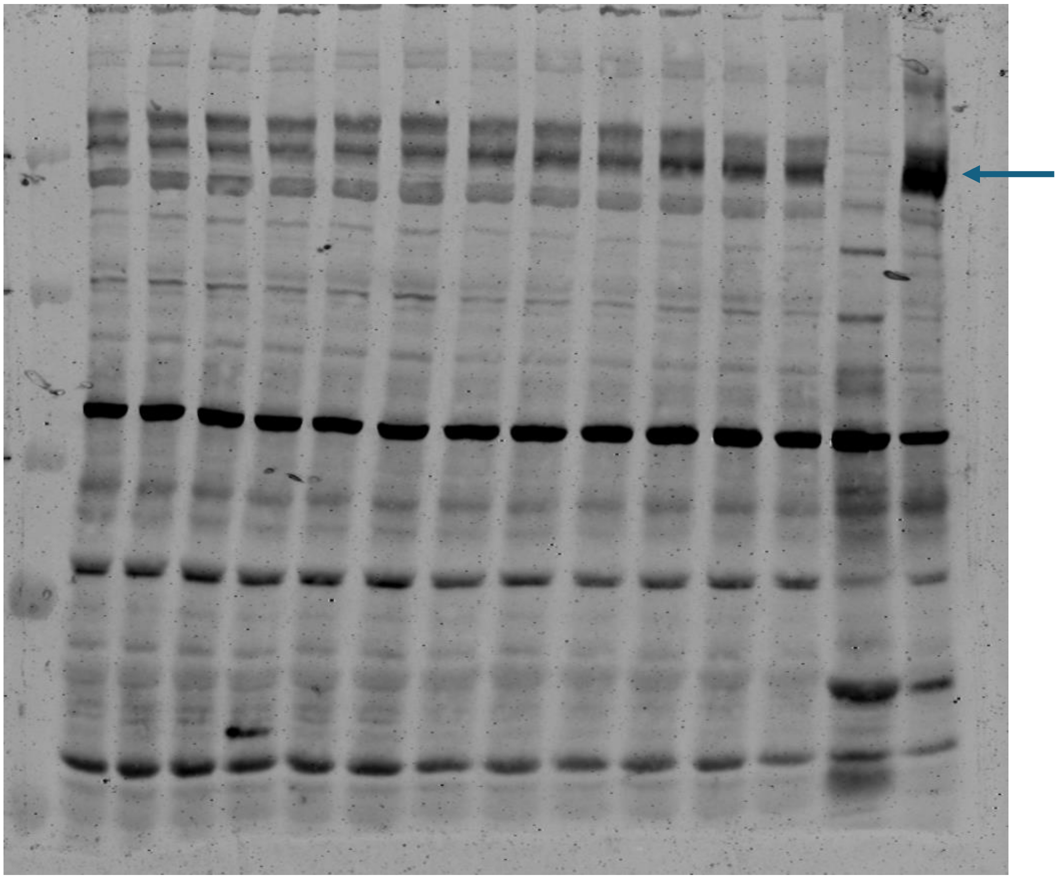

Vinculin (mouse)

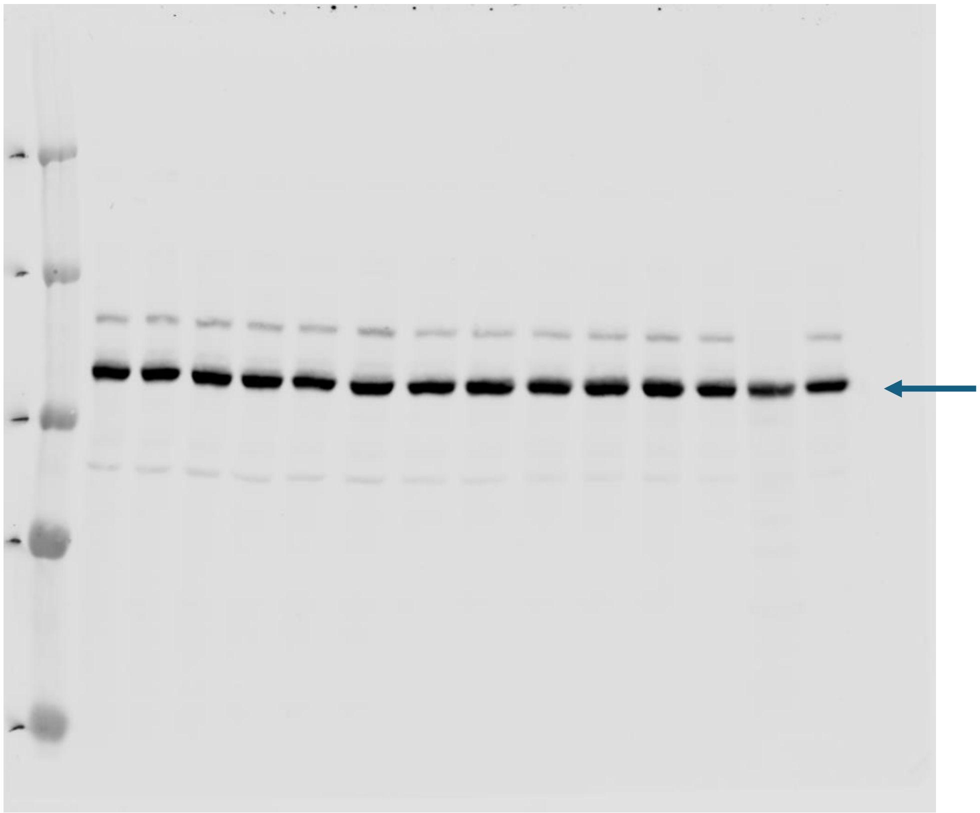

Dual channel

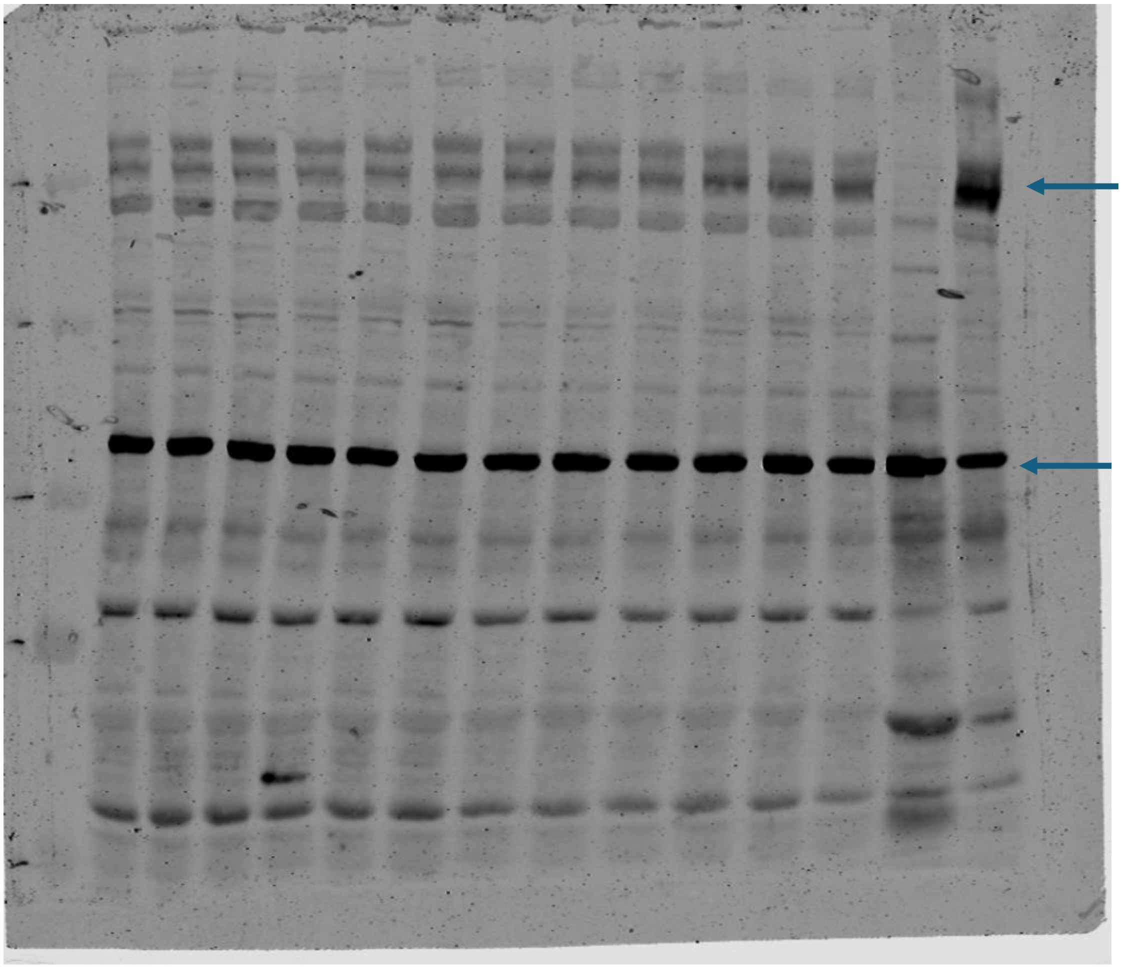

Figure S6. Figure 5B

ABCA1 (rabbit)

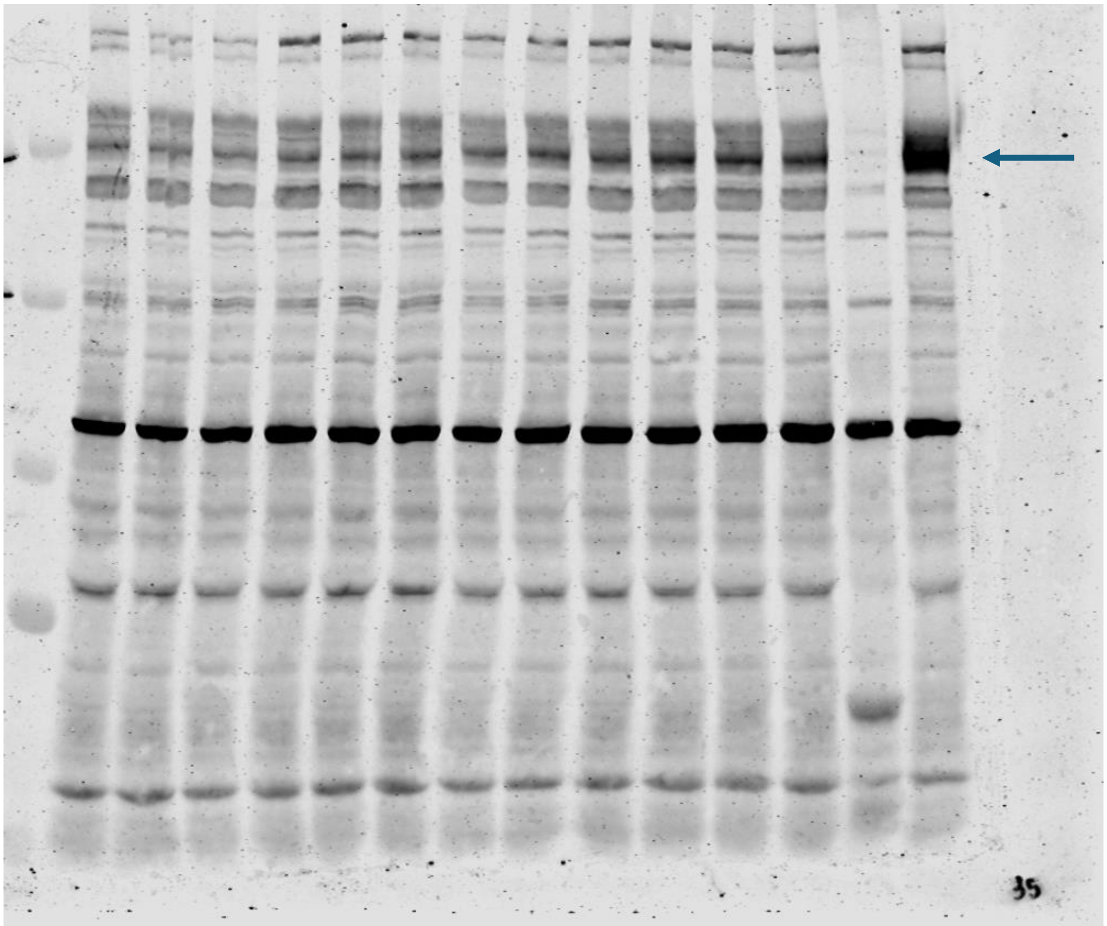

Vinculin (mouse)

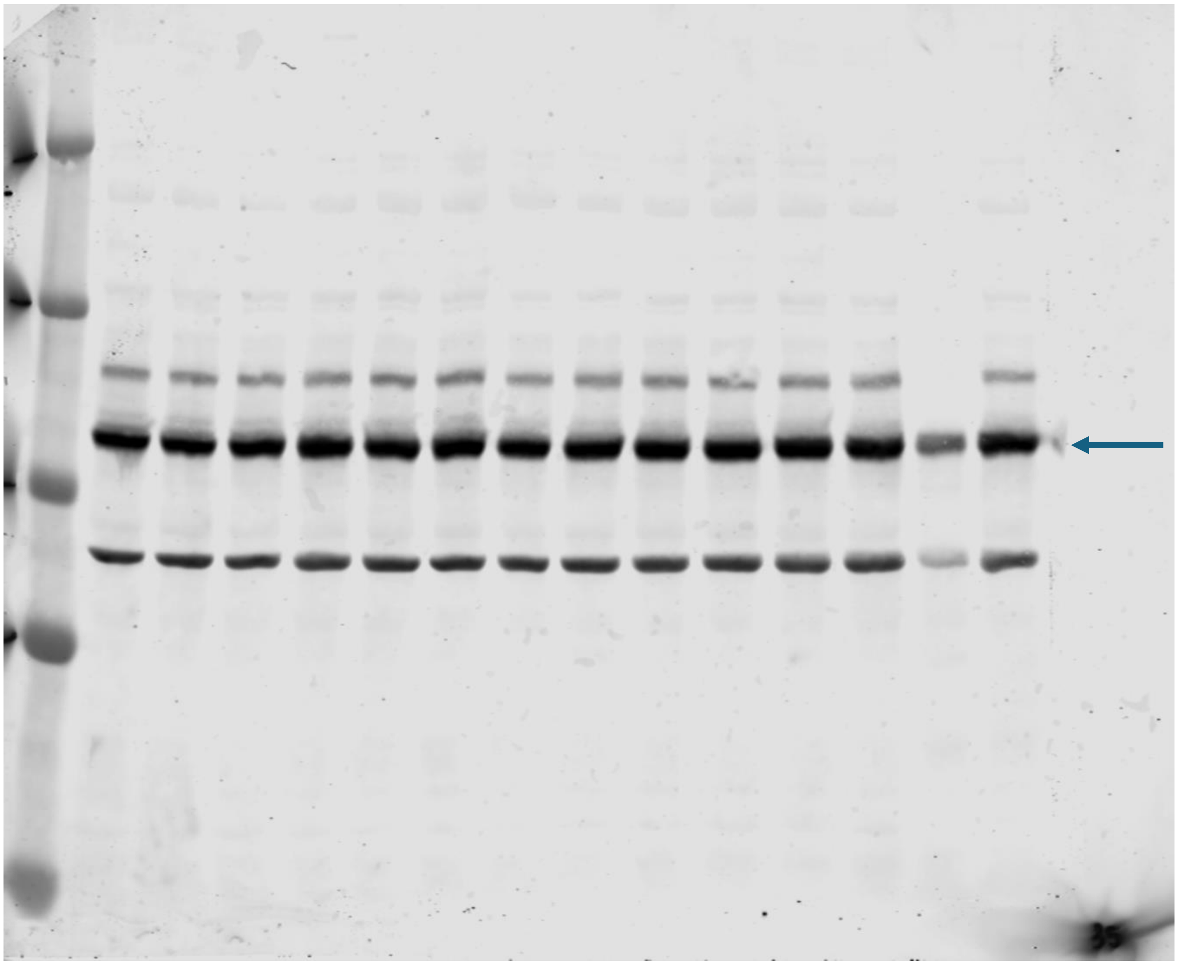

Dual channel

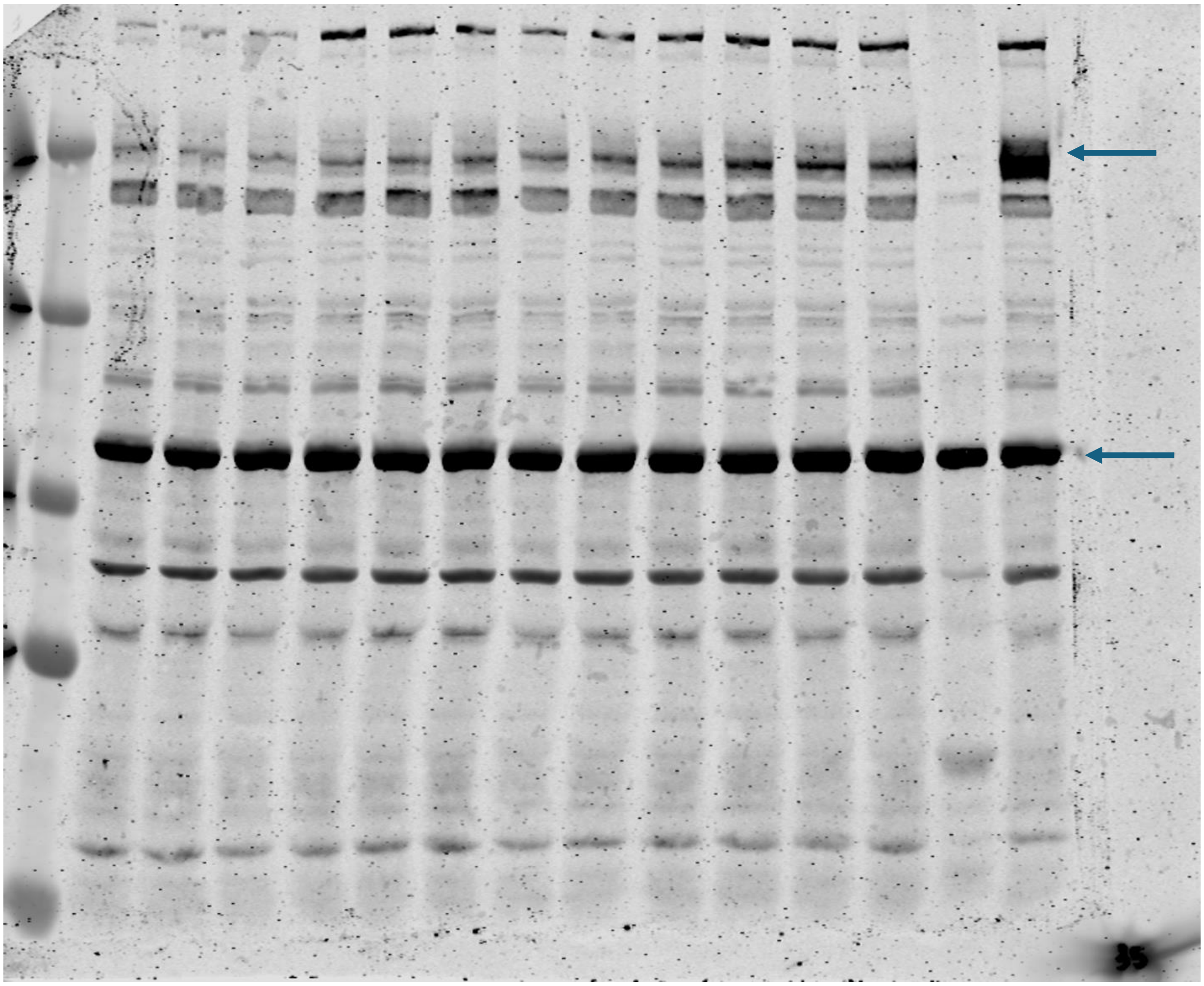

Figure S7. Figure 7B

ABCA1 (Rabbit channel)

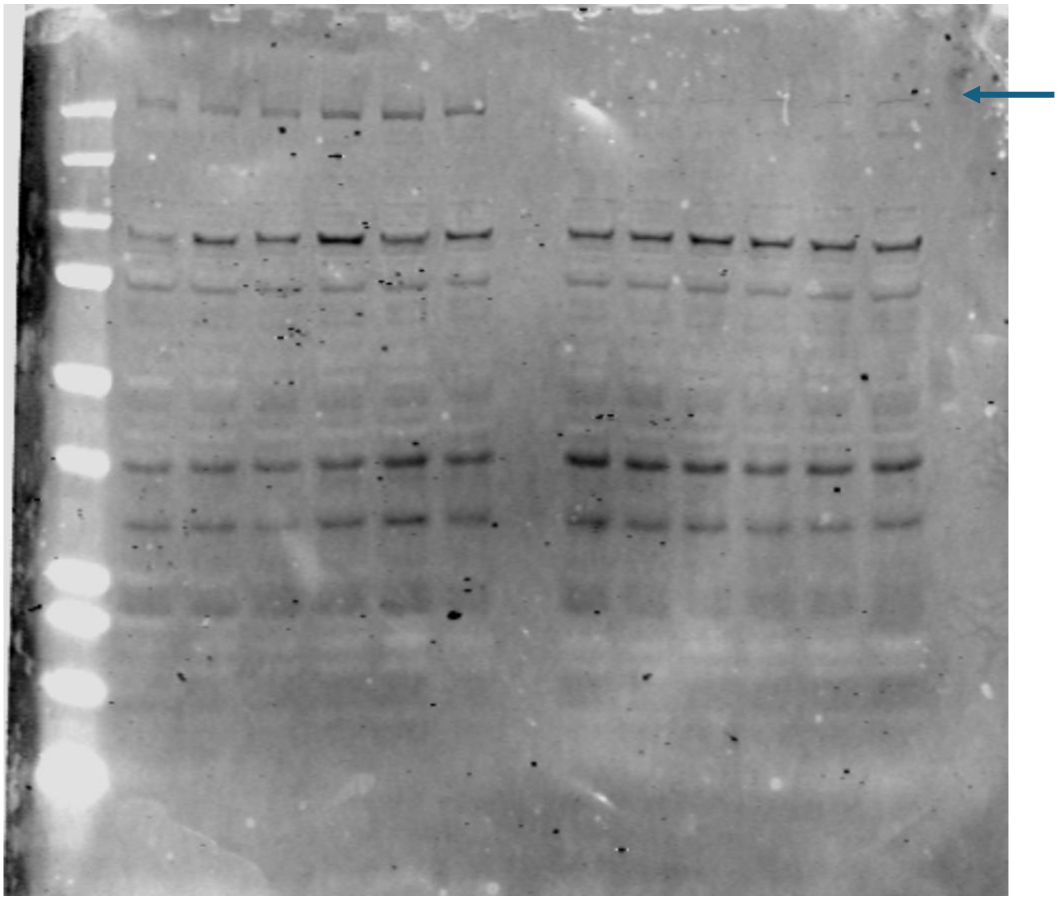

Vinculin (mouse channel)

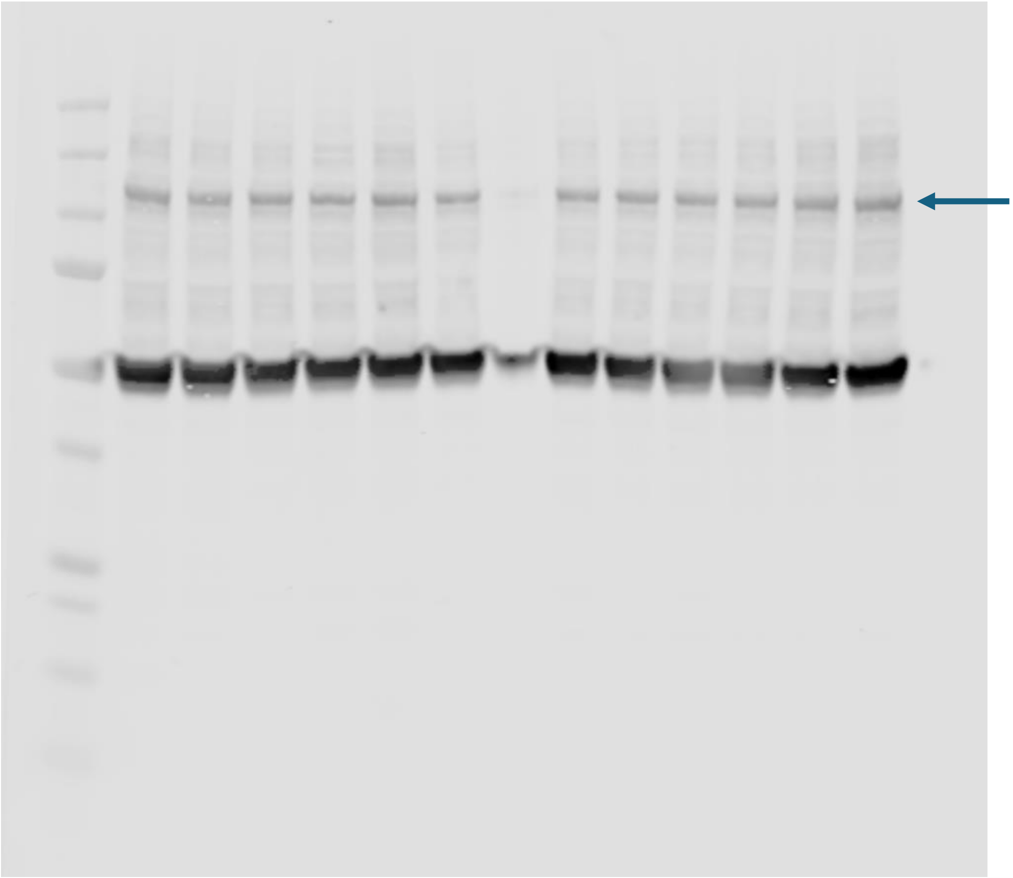

Dual channel

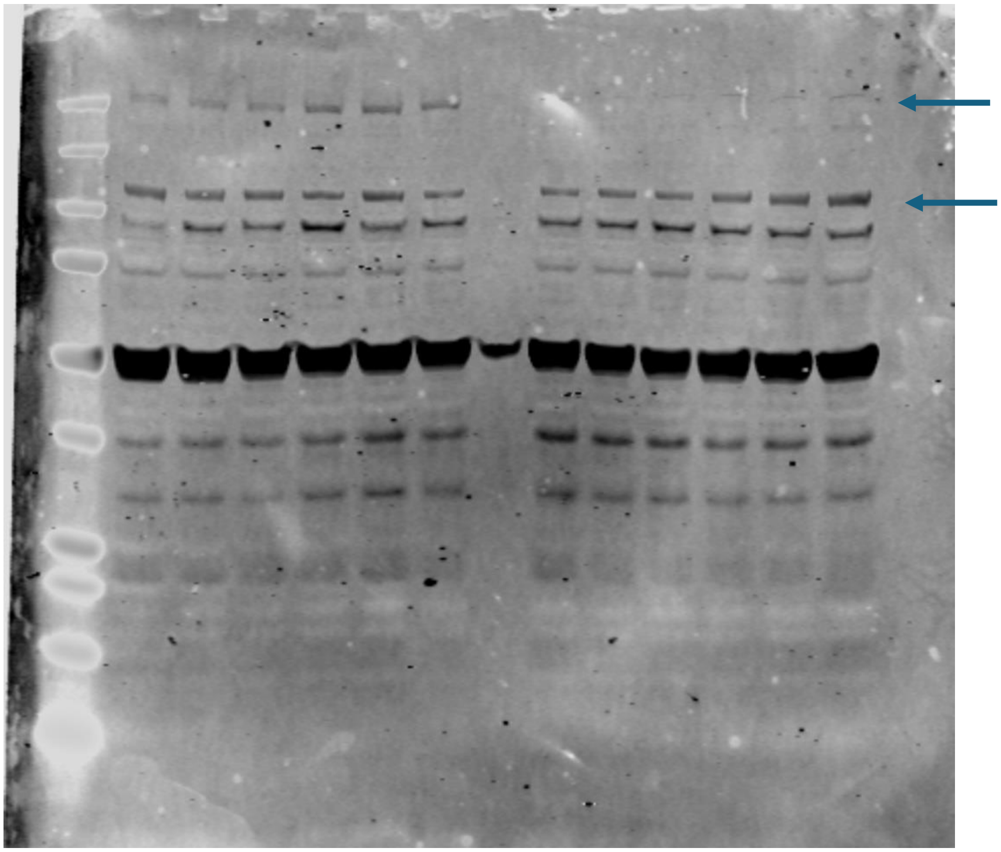

Supplement: Supplementary file 1 [file biomolecules-14-01301-s001.zip › biomolecules-3239098-supplementary.pdf]
